# Nutritional Status of Iodine and Association with Iron, Selenium, and Zinc in Population Studies: A Systematic Review and Meta-Analysis

**DOI:** 10.3390/nu17213432

**Published:** 2025-10-31

**Authors:** Sílvia Oliveira Lopes, Edna Miranda Mayer, Francilene Maria Azevedo, Aline Carrare Candido, Jersica Martins Bittencourt, Dayane de Castro Morais, Sylvia do Carmo Castro Franceschini, Silvia Eloiza Priore

**Affiliations:** 1 Department of Nutrition and Health, Federal University of Viçosa, Viçosa 36570-900, MG, Brazil; francilene.azevedo@ufv.br (F.M.A.); alinecarrare@hotmail.com (A.C.C.); jersica.cunha@ufv.br (J.M.B.); dayane.morais@ufv.br (D.d.C.M.); sylvia@ufv.br (S.d.C.C.F.); sepriore@ufv.br (S.E.P.); 2 Department of Agronomy, Federal University of Viçosa, Viçosa 36570-900, MG, Brazil; edna.mayer@ufv.br

**Keywords:** micronutrient deficiency, iodine, iron, selenium, zinc

## Abstract

Micronutrient deficiencies are not always present in individuals independently and may occur in association with other deficiency processes. Objective: Verify the association between the nutritional status of iodine and that of iron, selenium, and zinc in population studies. Methods: A bibliographic search was carried out in Medline, Web of Science, and CINAHL databases, without date and language restrictions, using English search terms and their synonyms. The search terms were joined by the Boolean operator AND while the respective synonyms were connected by OR following the PRISMA guidelines. Results: A total of 40 articles were included. The studies were published between 1993 and 2025, mostly involving both sexes and the main age groups were children and adolescents. Among the micronutrients analyzed, selenium stood out, being evaluated in 55.0% (*n* = 22) of the studies, followed by iron in 37.5% (*n* = 15) and zinc in 27.5% (*n* = 11). The most commonly used methods for assessing nutritional status were serum selenium, followed by urinary selenium. For iron, hemoglobin, ferritin, and serum iron were used in 73.3% (*n* = 11), 60.0% (*n* = 9), and 46.7% (*n* = 7) of the studies, respectively. For zinc, serum concentration was the most frequently used method; however, in one study, urinary zinc was evaluated. Overall, the nutritional status of iodine was associated with that of selenium, iron, and zinc, although this trend was not observed in some studies. Conclusions: The coexistence of deficiency processes in an individual still needs to be further elucidated. Combined strategies that effectively combat, prevent, and treat these micronutrient deficiencies must consider the possible interactions between them.

## 1. Introduction

Micronutrient deficiencies, especially of minerals such as iodine, iron, and zinc, are recognized as worldwide public health problems and are termed “Generalized Global Micronutrient Deficiencies”. At regional levels, selenium deficiency can be included among other deficiency processes [1,2].

Iodine deficiency is mainly monitored in school-age children, but it can affect individuals of all ages. Its nutritional status is influenced by dietary, socioeconomic, demographic, and lifestyle factors. Iodine is the micronutrient involved in the formation of thyroid hormones, triiodothyronine (T3) and Thyroxine (T4), which are essential for proper thyroid function. This micronutrient can be found in foods such as oysters, shellfish, and saltwater fish, and its concentration in foods may be affected by environmental factors [3].

Micronutrient deficiencies in an individual are not always present in isolation. In many cases, they occur in association with other deficiency processes. Therefore, understanding the metabolic pathways of mineral absorption (iodine, iron, selenium, and zinc) can enable the establishment of possible associations between deficiency processes. Also, the pathway of iodine absorption and formation of thyroid hormones can provide insight into the relationship between the nutrients covered in this systematic review. Iodine is highlighted because approximately 2 billion people worldwide suffer from iodine deficiency. Among adults, it can cause infertility, thyroid cancer, hypothyroidism, cognition deficiency, goiter, and reduced productivity, otherwise known as Iodine Deficiency Disorders (IDDs) [4,5].

Iron deficiency can impair oxygen transport and cognitive development, among other factors. Zinc plays a role in the immune response, inflammation, and oxidative stress control, which can increase an individual’s vulnerability to infectious diseases. Selenium’s antioxidant role is crucial for thyroid metabolism, working synergistically with iodine [6].

Thyroid hormone formation occurs on the apical surface of the cell. Iodide is oxidized by thyroperoxidase (TPO), an iron (Fe)-dependent enzyme, and in the presence of hydrogen peroxide (H_2_O_2_) to bind to thyroglobulin (Tg). Hydrogen peroxide is dangerous for thyrocyte and is therefore controlled by Glutathione Peroxidase (GPx), a selenoprotein (Se). The union of oxidized iodine with Tg generates the monoiodotyrosine (MIT) and diiodotyrosine (DIT) complexes. When MIT and DIT combine, triiodothyronine (T3) is formed, while the union of two DIT molecules forms Thyroxine (T4). For them to be expelled from the thyrocyte and for the inactive thyroid hormone (T4) to transform into the active hormone (T3), the presence of deiodinases I and II (DI and DII) is necessary, which are selenoproteins and also depend on zinc (Zn) for proper functioning [6].

Thus, iodine deficiency in association with other deficiency processes can cause a further decline in an individual’s health status. In relation to thyroid hormone formation, the contributions of minerals (iodine, iron, selenium, and zinc) and associated enzymes are very crucial.

In this context, this systematic review aimed to verify the association between the nutritional status of iodine and that of iron, selenium, and zinc in population studies.

## 2. Materials and Methods

The systematic review was conducted according to PRISMA guidelines—Preferred Reporting Items for Systematic Reviews and Meta-Analysis [7] and based on the following research question: “Is the nutritional status of iodine associated with that of iron, selenium, and zinc in population studies?” (Supplementary S1). Part of the content of this work, including data, analyses and figures, was presented in the doctoral thesis of Lopes, S.O [8].

### 2.1. Registration of Review

The systematic review was registered in the International Prospective Register of Systematic Reviews (PROSPERO) (CRD42021295475).

### 2.2. Inclusion and Exclusion Criteria

Inclusion criteria were human studies and population-based studies that investigated the correlation between iodine status and selenium, iron, or zinc status using biochemical assessments. There were no restrictions on gender, age, or study design. For longitudinal studies, baseline data were considered. There were no restrictions on the date, place, or language of publication.

Review studies and book chapters were excluded in addition to studies with pregnant women and subjects with genetic diseases such as Down syndrome, sickle cell anemia, HIV, and cancer (Supplementary S2).

### 2.3. Search and Selection of Articles

The search strategy followed the recommendations of the Peer Review of Electronic Search Strategies (PRESS) [9] (Supplementary S2). Three databases were used, Medline (via Pubmed), Web of Science, and CINAHL. Index terms and synonyms were selected with the aid of DECs and MESH terms coupled with Boolean operators OR for synonyms and AND for supplemental terms. The terms used were “iodine deficiency”, “iodine” with “famine, iron”, “anemia, iron-deficiency”, “growth disorders”, “iodine”, “malnutrition” (The full search strategy can be seen in Supplementary S2). The search for publications occurred on 2 August 2025. The search string used in Medline (via Pubmed) is available in the Supplementary Information (Supplementary S3).

### 2.4. Selection of Studies

The study selection was performed independently by a pair of researchers (SOL and EMM) using the Rayyan Software. Duplicates were identified and affected using the Rayyan software. Titles and abstracts were read first, followed by full articles. A third researcher (JMB) was asked to conduct an evaluation in the event of an impasse.

### 2.5. Data Extraction

Data extraction was performed independently by two researchers (SOL and EMM). The following data were extracted from articles into an Excel spreadsheet: author, year of publication, location, type of study, sample (number of participants, sex, and age), objective, method of assessing nutritional status of iodine and other micronutrients, association between iodine and other micronutrients, statistical test, *p*-value, and adjustment variables.

### 2.6. Evaluation of the Methodological Quality of the Selected Articles

To assess the risk of bias, tools recommended by the Joanna Briggs Institute were utilized, considering the study design and the following protocols: “Checklist for Analytical Cross-sectional Studies”, “Checklist for Case Control Studies”, “Checklist for Cohort Studies” and “Checklist for Randomized Controlled Trials” [10].

This instrument scores key aspects of each study: inclusion criteria, study description, exposure measurement, objectives, confounding factors, strategies used to tackle confounding factors, results measures, and statistical adequacy, among others, based on the study’s design [10].

The risk of bias was classified according to the percentage of affirmative responses (“yes”) being as follows: ≥70% considered low risk, between 50% and 69% moderate, and ≤49% high [11] (Supplementary S4). This assessment was not an exclusion criterion for articles in the review.

### 2.7. Data Synthesis and Analysis

Measures of correlation between the nutritional status of iodine (T3, T4, TSH, and UIC) and that of iron (serum iron, ferritin, and hemoglobin), selenium, and zinc were obtained from each study.

Meta-analysis was only performed on studies that had correlation. Adjustment for the calculations was considered. Age and sex stratification, when possible, did not influence the final result of the meta-analysis. The biomarkers used/type of analysis were organized according to the method used.

Data were loaded into an Excel spreadsheet and later exported to RStudio software, version 4.2.0, for meta-analysis. The metacor function of the meta package was used to summarize the correlation coefficients. To assess publication bias, the funnel symmetry test was applied, performed by the funnel function [12]. Heterogeneity among studies was assessed by Cochrane’s Q (χ^2^ *p* < 0.10) and quantified according to the proportion variance (I^2^). Values above 25%, 50%, and 75% were considered as low, moderate, and high heterogeneity, respectively. For all analyses, the random effect was considered, considering the moderate or high heterogeneity observed (I^2^ > 50%). All results are summarized in the forest plot graph, using the forest function of the metafor package [13]. To assess the impact of excluding each study individually, influence analysis (leave one out) was applied.

## 3. Results

**Figure 1:**
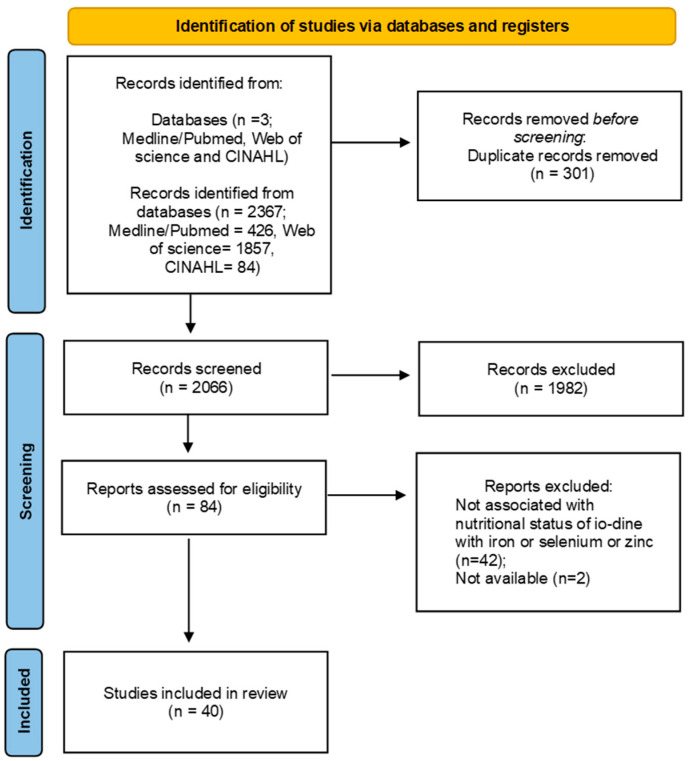
Scheme of the methodology adopted for the systematic review [7].

A total of 2367 articles were identified in three databases. After removing duplicates and reading titles, abstracts, and full texts, 40 articles were included in the systematic review (Supplementary S3). Figure 1 shows the selection processes. 

**Figure 2:**
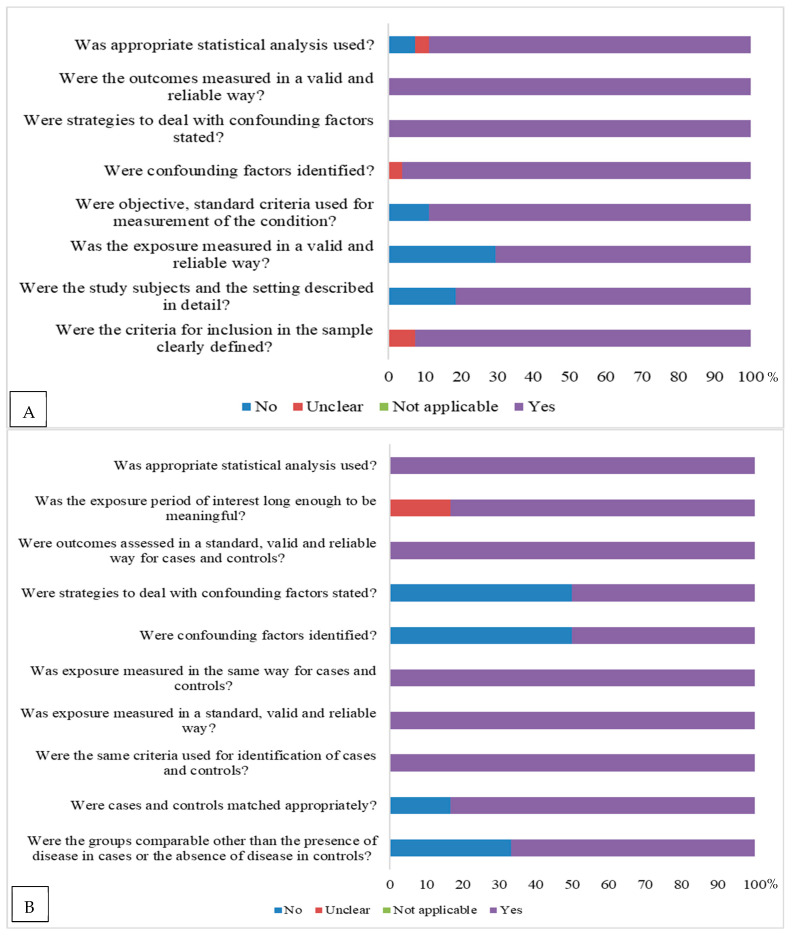

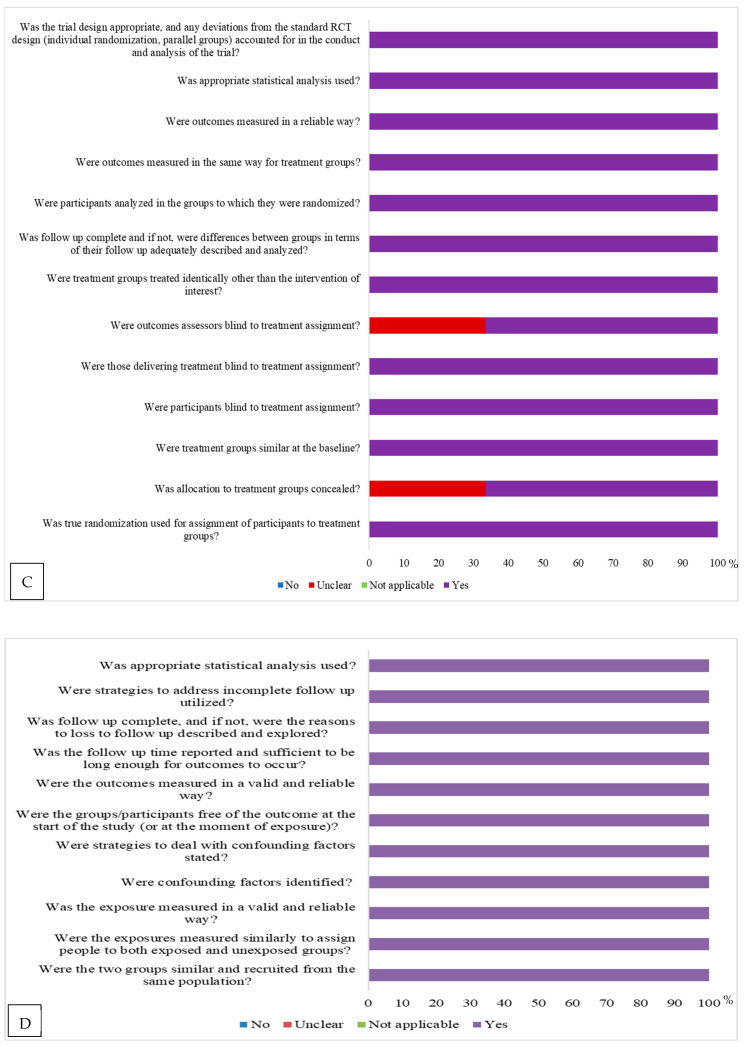
Risk of bias assessment according to the Joanna Briggs Institute’s risk of bias assessment tool (2020) [10] according to study design: (**A**) = for cross-sectional studies; (**B**) = control case; (**C**) = randomized clinical trial, and (**D**) = cohort.

Regarding the assessment of risk of bias, according to the study design, cross-sectional studies [14,15,16,17,18,19,20,21,22,23,24,25,26,27,28,29,30,31,32,33,34,35,36,37,38,39,40,41] presented a risk of bias. Studies with a control scheme [42,43,44,45,46,47] presented a risk of bias in relation to issues related to the control of confounding factors, and strategies to address confounding factors for other issues were considered to have a low risk of bias. In basic clinical trials [48,49,50] and cohorts [51,52,53], there was also a risk of bias (Figure 2).Among cross-sectional studies, 85.7% were classified as low risk, and the remainder as moderate risk. Among case–control studies, 83.3% were classified as low risk. All randomized clinical trials and cohort studies had a low risk of bias.

The studies were published between 1993 and 2025, with 70.0% (n = 28) [14,15,16,17,18,19,20,21,22,23,24,25,26,27,28,29,30,31,32,33,34,35,36,37,38,39,40,41], 15.0% (n = 6) [42,43,44,45,46,47], 7.5% (n = 3) [48,49,50], and 7.5% (n = 3) [51,52,53] corresponding to cross-sectional design, case–control, clinical trial, and cohort studies, respectively. In relation to sample composition, 15.0% (n = 6) [24,27,33,41,43,47] of the studies were conducted with women only, 2.5% (n = 1) [17] with men, and the remaining with both sexes. The countries with the highest number of works were Turkey [17,19,20,34,40,43,44,47] and Iran [21,22,24,27,28,29,30,32], with each country representing 20.0% (n = 8) of the studies. The age groups addressed in majority of the studies were children and adolescents 50.0% (n = 20), followed by adults and the elderly 32.5% (n = 13), with others 17.5% (n = 7) covering different ages (Table 1).

Table 2 presents the assessment methods of nutritional status and iodine association within iron, selenium, and zinc in population studies. The most used indicators for the direct or indirect assessment of nutritional status of iodine were as follows: Thyroid Stimulating Hormone (TSH) [14,15,17,18,19,21,22,24,25,26,27,29,30,33,34,35,36,37,38,39,41,42,43,44,45,48,49,50,52,53] and Thyroxine (T4) or free T4 [14,15,17,18,19,21,22,24,25,26,27,28,29,30,33,34,35,39,41,42,43,44,45,48,49,50,52,53] were utilized in 75.0% (n = 30) of the studies. The urinary iodine concentration (UIC) accounts for 62.5% (n = 25) [14,16,17,18,19,21,22,23,26,27,28,29,30,31,33,35,36,38,41,43,44,47,50,51,52], triiodothyronine (T3) represents -60.0% (n = 24) [14,15,17,19,21,22,24,25,26,33,34,35,36,37,41,42,43,44,45,48,50,51,53], ultrasonography and thyroid palpation account for 27.5% (n = 11) [14,19,20,25,26,31,33,40,43,44,51] and 20.0% (n = 8) [14,16,21,24,28,32,46,47], respectively. TSH, T3, and T4 are direct indicators of thyroid function, but also serve as indirect indicators of iodine nutritional status [54].

Among the micronutrients analyzed, selenium was most prominent, being evaluated in 55.0% (n = 22) [15,17,18,19,20,25,26,30,33,38,40,41,42,43,44,46,47,48,49,50,51] of the studies, iron 37.5% (n = 15) [14,21,22,23,29,31,33,34,35,36,37,39,45,49,52], and zinc 27.5% (n = 11) [16,17,23,24,27,28,32,40,42,45,47]. The nutritional status of selenium was mainly assessed through serum levels, however four articles evaluated urinary selenium [15,38,41,47] and Glutathione Peroxidase, reflecting selenium intake over 2 to 3 months [18,48,49]. To assess iron, 73.3% (n = 11) [14,22,23,31,34,35,37,39,45,52,53] of the articles employed hemoglobin, ferritin 60.0% (n = 9) [14,21,22,23,29,34,36,39,52], and serum iron 46.7% (n = 7) [14,34,35,36,37,39,45]. For zinc, serum level was mainly utilized, however a study employed urinary zinc [47] (Table 2).

TSH had both positive [14] and negative [35,37,42] correlations with hemoglobin. Furthermore, TSH presented a negative correlation with ferritin [22,37], serum iron [36,37,45,52], urinary iron [45], red blood cell count [52], and selenium [50]. T4 had a negative correlation with hemoglobin [14] and serum selenium [15,48]; it also had a positive correlation with hemoglobin [37,53], ferritin [22], serum iron [37], and transferrin saturation [37], and selenium [50] (Table 2).

**Figure 3:**
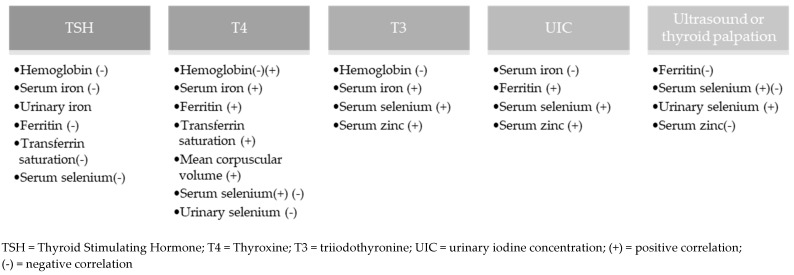
Relationship between methods for assessing nutritional status of iodine with selenium, iron, and zinc.

Figure 3 shows the markers related to the nutritional status of iodine and micronutrients. For 30.0% (n = 12) [15,18,22,23,24,25,27,30,31,32,33,43] of the studies, no statistical relationship was found between the nutritional status of iodine and selenium, iron, or zinc.

**Figure 4:**
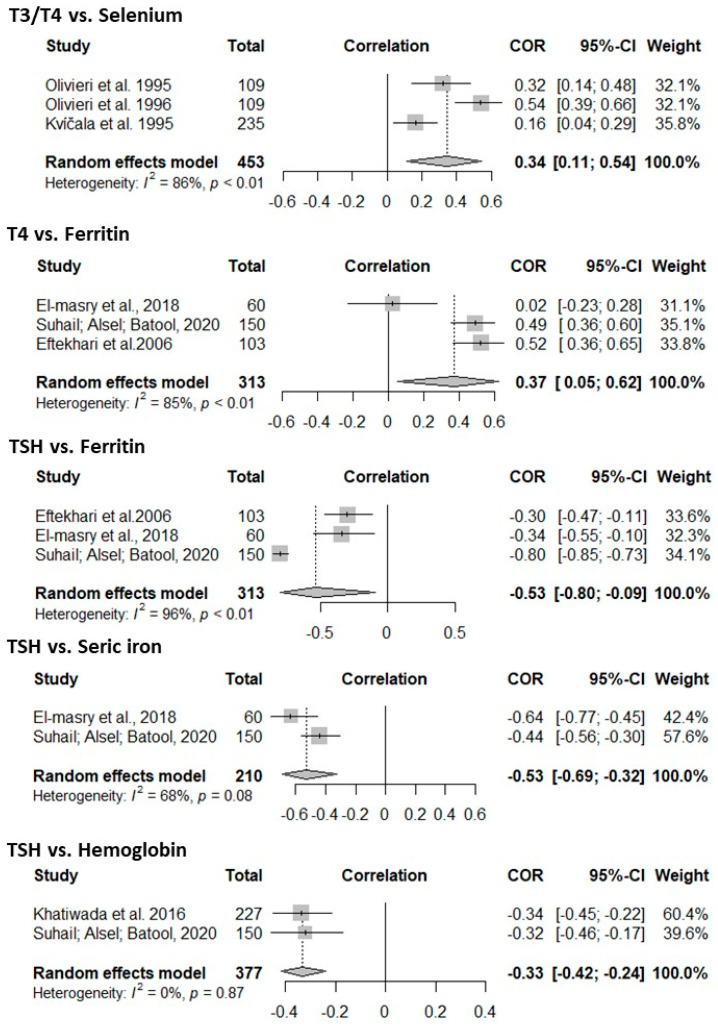
Meta-analysis of the correlation coefficients between the nutritional status of iodine (T3, T4, TSH, and UIC) and that of iron (serum iron, ferritin, and hemoglobin), selenium, and zinc. References [15,22,35,37,48,49,51].

Related to meta-analysis, there was a positive correlation between some markers of iodine nutrition and the evaluated micronutrients. For T3 and T4 there was a positive correlation with selenium [0.34 (0.11 to 0.54)], as well as the relationship between T4 and ferritin [0.37 (0.05 to 0.62)]. Also, there was a negative correlation between TSH and ferritin [−0.53 (−0.80 to −0.09)], serum iron [−0.53 (−0.69 to −0.32)], and Hemoglobin [−0.33 (−0.42 to −0.24)] (Figure 4). For the other markers of the nutritional status of iodine and other micronutrients, the correlation was not significant (Supplementary Figure S1).

Publication bias was observed due to the asymmetry of the funnel plots, as shown in Supplementary Figure S2. To assess the impact of excluding each study individually, influence analysis (leave one out) was applied (Supplementary Figure S3). For the meta-analysis assessing the correlation between T4 and ferritin, it was found that excluding one study would reduce heterogeneity (I2) to 0%. Similarly, in the correlation between TSH and ferritin, one of the studies contributed significantly to greater heterogeneity. For both, the methodological quality was assessed, and they were not excluded from the analysis.

## 4. Discussion

Micronutrient deficiencies are a health problem that must be addressed, especially because they can affect cognitive and motor development; additionally, in women of reproductive age, they can cause spontaneous abortions, fetal malformations, and other problems [54]. The causal factors for micronutrient deficiencies include low intake and/or absorption, the presence of diseases and changes in physiological status, such as pregnancy, breastfeeding, and varying micronutrient requirements by age [6].

Iodine nutritional status showed a positive correlation (direct or indirect) with T3/T4, selenium, and ferritin, and a negative correlation with ferritin, serum iron, and hemoglobin, as synthesized in the meta-analysis.

Low iodine intake can impair thyroid function and result in Iodine Deficiency Disorders (IDDs), goiter, and difficulties in cognitive development, among other problems. This micronutrient plays a role in the formation of the thyroid hormones T3 and T4, and deficiency processes can lead to decreased TSH levels and increased production of T3 compared to T4 [55].

Iodine is a chemical element absorbed primarily in the stomach and small intestine in the form of iodide. After absorption, most of it goes to the thyroid gland, where it participates in the synthesis of thyroid hormones T3 and T4. These hormones play a role in energy metabolism, growth and development, body temperature regulation, cardiac function, and central nervous system development. The negative feedback mechanism controls hormone production, ensuring balanced concentrations in the body [56].

The recommended daily iodine intake is 90 µg for preschool children (0 to 59 months), 120 µg for schoolchildren (6 to 12 years), 150 µg for adolescents (above 12 years) and adults, and 250 µg for pregnant and lactating women. The main biochemical marker for monitoring iodine nutritional status in populations is urinary iodine concentration, assessed by the median value. It is important to emphasize that this indicator applies to the population level, not the individual. The cutoff points are as follows: insufficient intake (≤99.0 µg/L), adequate (100–199 µg/L), more than adequate (200–299 µg/L), and excessive (≥300 µg/L), considering non-pregnant women. For pregnant women, <150 µg/L is considered insufficient, 150–249 is considered adequate, 250–499 µg/L is considered above requirements, and ≥500 µg/L is considered excessive [57].

The IUC marker may be influenced by the hydration status of the individual being analyzed, especially in random urine samples. However, the coefficient of variation is generally less than 10%. However, it is important to sample the population where this test is being performed to determine the varying levels of hydration in this group [57].

When iodine intake is insufficient, the thyroid gland activates adaptive mechanisms, such as increased TSH secretion, leading to hyperplasia and goiter formation. This process can maintain T3 production but compromises T4 levels, altering the individual’s metabolic function and overall health [57].

Excess iodine is also a significant health problem that must be addressed. High intake can cause bone changes, especially in menopausal women, and increase the risk of cardiovascular dysfunction associated with iodine-induced hyperthyroidism. The body has an adaptation mechanism to excess iodine, known as the Wolff–Chaikoff effect, in which there is a transient reduction in hormone synthesis through the inhibition of thyroid peroxidase (an iron-dependent enzyme). However, when this adaptation does not occur properly, clinical complications can arise [53,58].

Understanding the context of iodine deficiency or excess requires devising strategies. The main intervention strategy for preventing Iodine Deficiency Disorders (IDDs) is universal salt iodization, implemented worldwide since the 1990s. This measure is considered effective due to its low cost, broad coverage, and process safety. Iodine intake among the population can be classified as adequate in most cases, but in some countries, this remains a significant problem to be addressed. The continuous monitoring of iodine nutritional status is essential to prevent both deficiency and excess. Furthermore, process, impact, and sustainability indicators, such as assessment of urinary iodine concentration, goiter analysis, and monitoring of fortification policies, are essential to ensure the effectiveness of actions and protect the population’s health [59].

Some indicators used in the direct and indirect assessments of iodine nutritional status are UIC and assessment of thyroid function (TSH, TG, T3, T4, free T4), respectively. TSH is more sensitive for diagnosing iodine deficiency in newborns, although studies report difficulties in result interpretation [60]. In addition to these methods, the T3/T4 ratio can be mentioned, which also allows the inference of iodine nutritional status based on the proper functioning of the thyroid gland.

In newborns, as part of a physiological response, TSH peaks at birth, stimulating the production of the other thyroid hormones, T3 and T4. This assessment is considered a public health measure, as it can identify congenital hypothyroidism. This condition is one of the leading causes of intellectual disability, and if diagnosed early, it can help prevent permanent health effects [61]. In children and adolescents, TSH levels may remain higher than in adults and decrease with age, until they reach a “balance” and become more stable in adulthood [62]. The negative relationship between TSH and micronutrient status found in this review highlights the relationship between iron nutritional status and thyroid function [63] in different population groups.

Iodine deficiency, as well as other micronutrients, can be associated with food insecurity, making public health interventions important to help manage this. Because it has a cyclical effect, food insecurity can be caused by rising costs of healthy eating, low family education and income, and difficulties in accessing health services, among other factors. This contributes to an increased risk of nutritional deficiencies and, consequently, an increase in diseases associated with this deficiency [64].

Using iron deficiency anemia as an example, a study by Lopes et al. [64] found that individuals experiencing food insecurity were more likely to be anemic and have low ferritin levels, regardless of their level of insecurity. Thus, iron deficiency, in addition to causing anemia, can cause changes in iodine metabolism, since the thyroid hormone production pathway relies on this mineral for the action of peroxidase.

The interaction between micronutrient deficiencies is not well understood, which further reinforces the need for studies like this. One example is the heterogeneity of the studies included in this review, covering different population groups and the sample representativeness of the studies, as well as the difficulty in finding associations in humans [62]. Establishing associations is easier when using animal models [4]. It is known that deficiency processes can interact with each other because metabolic pathways are interrelated and adequate levels of micronutrients (iodine, iron, selenium, and zinc) are crucial for proper thyroid function [6].

Animal models have demonstrated the effect of selenium on iodine status and thyroid metabolism, but this relationship is not always confirmed when extrapolated to human studies. It is also suggested that combined iodine and iron interventions contribute to improved thyroid volume and function. Regarding zinc, there is limited evidence of its relationship with iodine status, although zinc is essential for proper thyroid function [65,66].

In an attempt to understand the relationship between iodine and selenium, iron, and zinc, hormonal production and the role of micronutrients in hormonal production pathways must be considered. Hormones are classified into three types: steroid hormones, tyrosine compounds, and peptide hormones. Iodine is a structural component of thyroid hormones.

In a study by Hortz et al. [66], combined iodine and selenium deficiency did not increase serum TSH concentrations in comparison to hypothyroidism. This finding was confirmed by other studies [67,68], in which partial selenium deficiency had no effect on hypothyroid symptoms. On the other hand [69,70], found higher TSH concentrations in selenium deficiency compared to isolated iodine deficiency. These discrepancies require further human studies to understand the coexistence of these processes. In addition to selenium and iron, zinc is a micronutrient needed for proper thyroid function. Studies in rats and humans have shown that zinc deficiency reduces iodothyronine levels, corroborating the findings of Ozata et al. [17], who found significantly lower zinc levels in people with endemic goiter, thus justifying a possible zinc–iodine relationship [71].

Many metabolic processes involved in human growth and development are under the direct or indirect control of thyroid hormones. The full functioning of thyroid hormones requires not only adequate levels of iodine but also other nutrients that are important for their formation. This review sought to encompass all biomarkers of the nutritional status for iodine and other nutrients (iron, selenium, and zinc). The age range and study design of the studies to be included were not restricted; furthermore, the review highlights the importance of not only assessing the nutritional status of one micronutrient but also considering other micronutrients that may be related to the deficiency process. This combined approach can contribute to effective programs to eradicate nutritional deficiencies.

One limitation of this review was the lack of restrictions on the publication period of the included studies, which resulted in the inclusion of work over 25 years old. This demonstrates that the discussion on the topic has spanned decades, but it also highlights that significant knowledge gaps remain. Despite the passage of time, new, well-designed research is still needed to deepen our understanding of this issue. Furthermore, understanding the interaction between micronutrient deficiency processes is essential, as synergism between different diseases can occur. A better understanding of these mechanisms, both individually and collectively, is crucial to guiding more effective health and nutrition interventions. Therefore, it is essential that new studies address this issue and contribute to the systematization of more consistent information.

## 5. Conclusions

The combination of strategies to help combat micronutrient deficiencies (iodine, iron, selenium, and zinc) must consider the possible interactions between them. There is a need to clarify the effects of iodine deficiency combined with other micronutrient deficiencies, the influence of food intake on the metabolism of thyroid hormones, and the best form of mineral supplementation to combat the established deficiency.

Furthermore, working with joint public policies to monitor and prevent these deficiencies is an important tool for maintaining the population’s health.

## Figures and Tables

**Table 1:** Description of the included studies organized according to study type and year of publication.

Author/Year	Place	Design	Sample (N; Sex; Age/Age Group)	Main Objective
Wolde-Gebriel et al., 1993 [14]	Shoa/Ethiopia	Cross-sectional	14,740; both sexes; 6–18 years	Examine the interrelations between three nutritional conditions (vitamin A, iodine, and iron deficiencies).
Kvíčala et al., 1995 [15]	Prague/Czech Republic	Cross-sectional	380; both sexes; 6–65 years	Evaluate the influence of selenium on the thyroid, hormonal parameters, and peripheral effects in selenium-deficient regions.
Hampel et al., 1997 [16]	Germany	Cross-sectional	5932; both sexes (3.692 women: 41 ± 17 years; 2.240 men: 39 ± 18 years)	Record goiter prevalence and iodine supply in Germany.
Ozata et al., 1999 [17]	Turkey	Cross-sectional	280; men; with goiter: n = 140 de 22.2 ± 0.19 aged; without goiter: n = 140 de 21.8 ± 0.28 aged	Determine the levels of iodine, zinc, selenium, and copper in patients with endemic goiter in order to investigate their role in the etiopathogenesis of endemic goiter in Turkey.
Zagrodzki et al., 2000 [18]	Poland	Cross-sectional	136; both sexes; 7–16 years	Investigate the role of selenium in iodine metabolism among children with goiter.
Erdoğan et al., 2001 [19]	Ancara, Kastamonu, Bayburt e Trabzon/Turkey	Cross-sectional	251 schoolchildren; both sexes; 9–11 years	Evaluate selenium level and thiocyanate (SCN-) overload and the possible contribution of this overload to endemic goiter and the thyroid hormone profile.
Aydin et al., 2002 [20]	Kayseri/Turkey	Cross-sectional	73 schoolchildren; both sexes; 7–12 years	Establish the effects of iodine and selenium levels on the size and functions of the thyroid gland in healthy schoolchildren.
Azizi et al., 2002 [21]	Iran	Cross-sectional	36,178 schoolchildren; both sexes; 8–10 years	Determine the relationship between serum ferritin and goiter, urinary iodine and thyroid hormones after iodine supplementation through iodized salt.
Eftekhari et al., 2006 [22]	Home/Iran	Cross-sectional	103 adolescents; women; 14–18 years	Determine thyroid hormone status in adolescent girls with iron deficiency.
Thurlow et al., 2006 [23]	Ubon Ratchathani/Thailand	Cross-sectional	567 children; both sexes; 6–13 years	Evaluate the prevalence of zinc and iodine deficiencies and their interrelations with vitamin A deficiency and anemia as well as associations with socioeconomic status, hemoglobin type, and anthropometry through a cross-sectional study.
Dabbaghmanesh et al., 2007 [24]	Marvdasht/Iran	Cross-sectional	1188; both sexes; 8–13 years	Determine the prevalence of zinc deficiency and current zinc status in schoolchildren with goiter.
Zagrodzki; Ratajczak, 2008 [25]	Poland	Cross-sectional	58; women; 23.57 ± 0.6 years	Identify parameters that characterize selenium status and secretion of sex hormones responsible for changes in indicators of thyroid function. In addition, reveal the correlation structure of parameters that express selenium status, sex hormone secretion, and thyroid function.
Doupis et al., 2009 [26]	Gyrocaster/Albania	Cross-sectional	112; both sexes; 52.8 ± 12.1 years	Study the thyroid status of people living in the Gyrocaster region of Southwestern Albania.
Keshteli et al., 2009 [27]	Isfahan/Iran	Cross-sectional	2330 schoolchildren; both sexes; 6–13 years	Estimate the prevalence of goiter and iodine status and investigate the role of selenium status as a possible contributor to endemic goiter among schoolchildren in Isfahan, 15 years after the start of a salt iodization program.
Moaddab et al., 2009 [28]	Semirom/Iran	Cross-sectional	1828; both sexes; schoolchildren	Associate goiter with serum zinc in a mountainous region of Iran.
Hashemipour et al., 2010 [29]	Isfahan/Iran	Cross-sectional	2331 schoolchildren; both sexes; 6–13 years	Estimate the prevalence of goiter and iodine status and investigate the role of iron deficiency as a possible contributor to endemic goiter in schoolchildren of Isfahan, 15 years after the start of a salt iodization program.
Keshteli et al., 2010 [30]	Isfahan/Iran	Cross-sectional	2331 schoolchildren; both sexes; 6–13 years	Determine the association between zinc status and goiter in schoolchildren of Isfahan.
Henjum et al., 2011 [31]	Algeria	Cross-sectional	394; women; 15–45 years	Assess iodine status (thyroid volume and urinary iodine concentration) and its determinants in Saharawi refugee women.
Sanjari; Gholamhoseiniana; Nakhaee, 2012 [32]	Kerman/Iran	Cross-sectional	5380 schoolchildren; both sexes; 8–12 years	Determine the difference in serum zinc levels between children with and without goiter aimed at finding evidence on the probable role of zinc deficiency in endemic goiter.
Liu et al., 2013 [33]	Chengdu/China	Cross-sectional	1205; both sexes; 18 years or older	Investigate the relationship between selenium level, thyroid volume, and goiter in an area with sufficient iodine.
Yavuz et al., 2014 [34]	Turkey	Cross-sectional	330; both sexes; ±14 years	Evaluate the effect of iron level on thyroid hormone profile in adolescents living in a mild iodine-deficient area in Turkey
Khatiwada et al., 2016 [35]	Sankhuwasabha e Dhankuta/Nepal	Cross-sectional	227 schoolchildren; both sexes; 6–12 years	Investigate the association between iron level and thyroid function among Nepalese children living in mountainous regions.
Luo et al., 2017 [36]	United States of America	Cross-sectional	7672; both sexes; 20 years or older	Investigate the combined association of serum iron and urinary iodine concentrations with serum thyroid hormone measurements using a national representative sample from the National Health and Nutrition Examination Survey (NHANES).
Suhail; Alsel.; Batool, 2020 [37]	Saudi Arabia	Cross-sectional	150; both sexes; 24–76 years	Estimate the prevalence and association of thyroid dysfunction with anemia/body iron status in the Saudi population of the northern border.
Campos et al., 2021 [38]	Bahia/Brazil	Cross-sectional	982 schoolchildren; both sexes; 6–14 years	Evaluate the nutritional status of selenium and iodine in schoolchildren.
Islam et al., 2021 [39]	Bangladesh	Cross-sectional	405; women; 0–81 years	Inspect the correlation between iron deficiency anemia and thyroid disorders in Bangladeshi women.
Turan; Turksoy, 2021 [40]	Yozgat/Turkey	Cross-sectional	181 (98 with goiter and 83 without goiter); both sexes; 18–65 years	Compare serum levels of trace elements, such as selenium, zinc, and copper in patients with euthyroid nodular goiter and healthy participants.
Berger et al., 2025 [41]	New Zealand	Cross-sectional	37; women; 40–63 years	Investigate the effect of low bread intake on iodine and selenium intake and status, and thyroid function in mid-life women in New Zealand.
Ravaglia et al., 2000 [42]	Bolonha, Emilia Romagna/Italy	Control case	132; both sexes; control: aged 20–64 years; elderly group: n = 44 aged 65–89 years; oldest-old group: n = 44 aged 90–107 years (24 were 100 years old or older)	Evaluate the relationships between thyroid function and blood levels of selenium, zinc, retinol, and alpha-tocopherol in a selected group of healthy free-living Italian subjects.
Cinaz et al., 2004 [43]	Turkey	Control case	905; both sexes; 6–12 years	Investigating the prevalence of goiter, serum selenium, and urine iodine status among school children in the Ankara region of Turkey.
Hekimsoy et al., 2004 [44]	Turkey	Control case	102; both sexes; with goiter n = 72 aged 43 ± 11 years; without goiter: n = 30 aged 40.6 ± 13.6 years	Measure iodine excretion rates in patients with diffuse or nodular goiter and examine plasma selenium concentrations in order to verify whether selenium deficiency may be related to the genesis of goiter in the studied region.
Kandhro et al., 2008 [45]	Sindh/Pakistan	Control case	186; women; with goiter: n = 69 without goiter: n = 117; 21–45 years	Evaluate iron concentration in biological samples (serum and urine) and other biochemical parameters such as TSH, free triiodothyronine, and free Thyroxine in goiter patients and compare them with healthy women of the same age residing in the same area.
Kishosha; Galukand; Gakwaya 2011 [46]	Uganda	Control case	92; both sexes; 18–35 years	Determine serum selenium levels in goiter patients and non-goiter controls and determine the association between goiter and selenium levels in these patients.
Çelik et al., 2014 [47]	Hatay/Turkey	Control case	214 schoolchildren; both sexes; 6–12 years	Investigate urine iodine, selenium, zinc, copper, or molybdenum deficiencies in children aged 6 to 12 years in two schools in the Hatay province.
Olivieri et al., 1995 [48]	Vicenza/Italy	Randomized clinical trial	109; both sexes; Group I: n = 36 adults aged 20–44 years Group II: n = 36 individuals aged 20–44 years; Group III: n = 37 elderly aged 65 years and older	Investigate the relationships between age, selenium status, and thyroid hormones in three groups of healthy free-living individuals of different ages, paired by sex distribution.
Olivieri et al., 1996 [49]	Vicenza/Italy	Randomized clinical trial	109; both sexes; Group I: n = 36 adults aged 20–44 years Group II: n = 36 individuals aged 20–44 years; Group III: n = 37 elderly aged 65 years and older	Investigate the relationships between age, selenium levels, and thyroid hormones in three groups of healthy free-living individuals of different ages, paired by sex distribution. Further, measure serum and erythrocyte zinc to assess possible interactions with circulating levels of thyroid hormones.
Gashu et al., 2009 [50]	Amhara/Ethiopia	Randomized clinical trial	624; both sexes; 6–60 months	Investigate the influence of selenium inadequacy on thyroid response in children.
El-masry et al., 2018 [51]	Assiut e Qena/Egypt	Clinical trial	805; both sexes; women aged 18–22, 40–45 and 60–65 years; men aged 60–65 years	Study the associations between serum selenium concentration, thyroid volume, and the risk of thyroid gland enlargement in an area with mild iodine deficiency before and after the introduction of iodine fortification.
Rasmussen et al., 2011 [52]	Denmark	Cohort	120; (60 anemic and 60 non-anemic); both sexes; 2–16 years	Investigate the possible occurrence of thyroid dysfunction among children with isolated iron deficiency anemia of various severities and test whether oral iron replacement therapy alone can reverse the associated thyroid function disorders, and if additional or present therapies are needed.
Gu et al., 2019 [53]	Tianjin/China	Cohort	12,310; both sexes; 45.5 (±11.4) years	Examine whether thyroid hormones under physiological conditions can affect the development of anemia in the general population

**Table 2:** Methods employed for the assessment of nutritional status and association of iodine, iron, selenium, and zinc in population studies.

Author/Year	Method Evaluation		Association Between Nutritional Status of Iodine and Micronutrients
	Nutritional Status of Iodine	Micronutrient (Method Used)	
Wolde-Gebriel et al., 1993 [14]	UIC; TSH; T3; T4; Thyroxine-Binding Globulin; thyroid palpation	Hemoglobin; ferritin; serum iron; total iron binding capacity; transferrin; hematocrit; mean corpuscular volume	Negative correlation between T3, T4, and Thyroxine Binding Globulin with hemoglobin.
Kvíčala et al., 1995 [15]	UIC; TSH; T3; T4; thyroid ultrasound	Serum selenium; urinary selenium; urinary selenium/creatinine; and creatinine concentration (hair)	Positive correlation between T3 and serum selenium and selenium/creatinine; positive correlation between thyroid volume, serum selenium, and urinary selenium; positive correlation between T4/T3 and serum selenium; negative correlation between thyroid volume and selenium/creatinine; negative correlation between T4, serum selenium and urinary selenium.
Olivieri et al., 1995 [48]	TSH; T3; T4	Serum selenium and erythrocyte Glutathione Peroxidase	Negative correlation between T4, positive T3/T4, and selenium (all population). In older individuals, negative with T4 and positive T3/T4.
Olivieri et al., 1996 [49]	TSH; T3; T4	Serum selenium; Glutathione Peroxidase; serum zinc; and red blood cells	Positive correlation in the older individual’s group between T3/T4, selenium status, and Glutathione Peroxidase.
Hampel et al., 1997 [16]	UIC; thyroid palpation	Serum zinc	NA
Ozata et al., 1999 [17]	UIC; TSH; T3; T4	Serum zinc; selenium; and copper	UIC and zinc were lower in the group with goiter than in the group without goiter.
Ravaglia et al., 2000 [41]	TSH; T3; T4	Selenium and serum zinc	Positive correlation between T3 and T3/T4 with zinc in individuals under 90 years of age.
Zagrodzki et al., 2000 [18]	UIC; TSH; T4	Serum selenium and Glutathione Peroxidase	Lower selenium concentrations and Glutathione Peroxidase activity in the group with goiter compared to the group without goiter.
Erdoğan et al., 2001 [19]	UIC; TSH; T3; T4; thyroid ultrasound; thyroglobulin	Serum selenium	NA
Aydin et al., 2002 [20]	Thyroid ultrasound	Serum selenium	Positive correlation between thyroid volume and selenium; selenium and iodine levels in children with goiter were lower.
Azizi et al., 2002 [21]	UIC; TSH; T3; T4; thyroid palpation	Ferritin	Lower goiter rates in children with higher ferritin concentrations.
Cinaz et al., 2004 [42]	UIC; TSH; T3; T4; Anti-TPO; thyroid ultrasound	Serum selenium	Selenium level was lower in goiter group.
Hekimsoy et al., 2004 [43]	UIC; TSH; T3 and T4; total and free; thyroid ultrasound	Serum selenium	NA
Eftekhari et al., 2006 [22]	UIC; TSH; T3; T4; T3 and T4 free; uptake of triiodothyronine resin; reverse triiodothyronine concentrations	Hemoglobin; ferritin; total iron binding capacity; serum selenium	Positive correlation between T4, negative TSH, negative T3/T4 with ferritin; ferritin contributed to T3 concentration.
Thurlow et al., 2006 [23]	UIC	Serum zinc; hemoglobin; and ferritin	NA
Dabbaghmanesh et al., 2008 [24]	TSH; T3 and T4 free; thyroid palpation	Serum zinc	NA
Kandhro et al., 2008 [44]	TSH; T3; T4	Hemoglobin; serum and urinary iron; transferrin receptor; mean corpuscular volume; mean corpuscular hemoglobin concentration; zinc protoporphyrin	Serum and urinary iron concentrations were reduced in the group with goiter and increased TSH.
Zagrodzki; Ratajczak, 2008 [25]	TSH; T3; T4 Plasma Glutathione Peroxidase; anti-TPO; thyroid ultrasound	Serum selenium	NA
Doupis et al., 2009 [26]	UIC; TSH; T3; T4 liver; anti-TPO; anti-TG; thyroid ultrasound	Serum selenium	NA
Gashu et al., 2009 [49]	UIC; TSH; T3; T4; thyroglobulin	Serum selenium	Positive correlation between T3 and T4, and negative correlation between TSH, thyroglobulin, and selenium. Despite adequate iodine status, children with low selenium levels had lower T3 and T4 and higher TSH concentrations.
Keshteli et al., 2009 [30]	UIC; TSH; T4; anti-TPO; anti-TG	Serum selenium	Mean serum selenium in children with and without goiter were different. The prevalence of selenium deficiency was higher in boys and girls with goiter than without goiter.
Moaddab et al., 2009 [28]	UIC; T4; Thyroid palpation	Serum zinc	NA
Hashemipour et al., 2010 [29]	UIC; TSH; T4; anti-thyroglobulin; anti-thyroperoxidase	Ferritin	Positive correlation between UIC and ferritin.
Keshteli et al., 2010 [27]	UIC; TSH; T4	Serum zinc	Mean serum zinc levels in children with and without goiter were different.
Kishosha; Galukand; Gakwaya 2011 [45]	Thyroid palpation	Serum selenium	Selenium levels between the populations with and without goiter were different; selenium levels above 102.8 μg/L presented a protective effect against goiter.
Rasmussen et al., 2011 [50]	UIC; thyroid ultrasound	Serum selenium	Serum selenium concentration was associated with the enlargement of the thyroid gland.
Henjum et al., 2012 [31]	UIC; thyroid ultrasound	Hemoglobin	NA
Sanjari; Gholamhoseiniana; Nakhaee, 2012 [32]	Thyroid palpation	Serum zinc	NA
Liu et al., 2013 [33]	UIC; TSH; T3; T4 livers; anti-TPO; anti-TG; thyroid ultrasound	Serum selenium	NA
Çelik et al., 2014 [46]	UIC; thyroid palpation	Urinary zinc and selenium	Positive correlation between selenium and zinc with iodine.
Yavuz et al., 2014 [34]	TSH; T3 and T4 free	Hemoglobin; ferritin; serum iron; mean corpuscular volume; and total serum iron binding capacity	NA
Khatiwada et al., 2016 [35]	UIC; TSH; T3; T4	Hemoglobin; serum iron; total iron binding capacity; transferrin saturation	Mean TSH level was higher in the anemic; T3 was higher in children with sufficient iron; serum iron level and transferrin saturation were different in iodine-deficient and iodine-sufficient children. Negative correlation between TSH, transferrin saturation, and hemoglobin; the risk of having hypothyroidism in anemic and iron-deficient children was 5.513 and 1.939, respectively, compared to non-anemic and iron-sufficient children.
Luo et al., 2017 [36]	UIC; TSH; T3; T4; T3 and T4 free	Serum iron	When serum iron concentrations were normal, a high urinary iodine concentration was associated with reduced free T3 and increased risk of elevated TSH. When serum iron and iodine levels were low, there was an association with reduced free T3 level and increased TSH level.
El-masry et al., 2018 [51]	UIC; TSH; T3; T4	Hemoglobin; ferritin; hematocrit; red blood cell count; mean corpuscular volume; mean corpuscular hemoglobin; red blood cell distribution width; iron concentration; total iron binding capacity; transferrin saturation; and iron binding capacity	Positive correlation between T3 and negative TSH with serum iron; positive between TSH and red cell distribution width; positive T3 and red blood cell count; negative between TSH with hemoglobin, hematocrit, and red blood cell count.
Gu et al., 2019 [52]	TSH; T3 and T4 free	Hemoglobin	Increased T3 and T4 concentrations were associated with decreased incidence of anemia. There was an association between T3, T4, and annual changes in hemoglobin.
Suhail; Alsel; Batool, 2020 [37]	TSH; T3; T4	Hemoglobin; Ferritin; Serum iron; transferrin saturation; mean corpuscular volume; mean corpuscular hemoglobin	TSH has a negative correlation with hemoglobin, serum iron, ferritin, transferrin saturation, mean corpuscular volume, and mean corpuscular hemoglobin; T4 has a positive correlation with hemoglobin, serum iron, ferritin, transferrin saturation, mean corpuscular volume, and mean corpuscular hemoglobin.
Campos et al., 2021 [38]	UIC; TSH	Urinary selenium	Positive correlation between UIC and selenium.
Islam et al., 2021 [39]	TSH; T4	Hemoglobin; ferritin; serum iron; total iron binding capacity	Positive correlation between hypothyroidism and iron deficiency anemia (IDA); association between congenital hypothyroidism and IDA.
Turan; Turksoy, 2021 [40]	Thyroid ultrasound	Serum selenium and zinc	Serum zinc and selenium levels were higher in individuals without goiter compared to those with goiter.
Berger et al., 2025 [41]	UIC; TSH; T3 free; T4 free, TG; anti-TPO; anti-TG	Plasma and urinary selenium	Plasma selenium and urinary iodine excretion as predictors of the T3:T4 ratio were significant in explaining 13% of the model.

UIC = urinary iodine concentration; TSH = Thyroid Stimulating Hormone; T4 = Thyroxine; T3 = triiodothyronine; TPO = thyroid peroxidase; TG = thyroglobulin; NA = there was no association between iodine’s nutritional status and evaluated micronutrients (*p* > 0.05).

## Data Availability

Analyses are available in the Supplementary Materials and information was taken from articles included in this review and referenced in the analyses.

## Supplementary Materials

The following supporting information can be downloaded at: https://www.mdpi.com/article/10.3390/nu17213432/s1, Supplementary S1. Prisma 2020 Checklist; Supplementary S2. Peer review of electronic search strategies; Supplementary S3. Search strategies in the databases used; Supplementary S4. Risk of bias for each individual study assessed by the Joanna Briggs Institute critical appraisal checklist for studies; Supplementary Figure S1. Meta-analysis of the correlation coefficients between the nutritional status of iodine (T3, T4, TSH, and UIC) and that of iron (serum iron, ferritin and hemoglobin), selenium and zinc; Supplementary Figure S2. Funnel plot of meta-analysis of the correlation coefficients between the nutritional status of iodine (T3, T4, TSH, and UIC) and that of iron (serum iron, ferritin and hemoglobin), selenium and zinc; Supplementary Figure S3. Influence analysis was applied.
