# Supplementary material for: Nutritional Status of Iodine and Association with Iron, Selenium, and Zinc in Population Studies: A Systematic Review and Meta-Analysis
Source: Nutrients. 2025 Oct 31;17(21):3432. doi: 10.3390/nu17213432 (PMC12608380; doi:10.3390/nu17213432)
Supplement: Supplementary file 1 [file nutrients-17-03432-s001.zip › nutrients-3870440-supplementary.pdf]

Supplementary 1. PRISMA 2020 Checklist

Section and Topic	Item #	Checklist item	Location where item is reported
**TITLE**			
Title	1	Identify the report as a systematic review.	2-3
**ABSTRACT**			
Abstract	2	See the PRISMA 2020 for Abstracts checklist.	25
**INTRODUCTION**			
Rationale	3	Describe the rationale for the review in the context of existing knowledge.	46-61
Objectives	4	Provide an explicit statement of the objective(s) or question(s) the review addresses.	62-63
**METHODS**			
Eligibility criteria	5	Specify the inclusion and exclusion criteria for the review and how studies were grouped for the syntheses.	72-80
Information sources	6	Specify all databases, registers, websites, organisations, reference lists and other sources searched or consulted to identify studies. Specify the date when each source was last searched or consulted.	81-90 and Supplementary 2.
Search strategy	7	Present the full search strategies for all databases, registers and websites, including any filters and limits used.	Supplementary 1 and 2
Selection process	8	Specify the methods used to decide whether a study met the inclusion criteria of the review, including how many reviewers screened each record and each report retrieved, whether they worked independently, and if applicable, details of automation tools used in the process.	92-94
Data collection process	9	Specify the methods used to collect data from reports, including how many reviewers collected data from each report, whether they worked independently, any processes for obtaining or confirming data from study investigators, and if applicable, details of automation tools used in the process.	95-100
Data items	10a	List and define all outcomes for which data were sought. Specify whether all results that were compatible with each outcome domain in each study were sought (e.g. for all measures, time points, analyses), and if not, the methods used to decide which results to collect.	95-100
	10b	List and define all other variables for which data were sought (e.g. participant and intervention characteristics, funding sources). Describe any assumptions made about any missing or unclear information.	95-100
Study risk of bias assessment	11	Specify the methods used to assess risk of bias in the included studies, including details of the tool(s) used, how many reviewers assessed each study and whether they worked independently, and if applicable, details of automation tools used in the process.	101-112
Effect measures	12	Specify for each outcome the effect measure(s) (e.g. risk ratio, mean difference) used in the synthesis or presentation of results.	114-124
Synthesis methods	13a	Describe the processes used to decide which studies were eligible for each synthesis (e.g. tabulating the study intervention characteristics and comparing against the planned groups for each synthesis (item #5)).	73-79
	13b	Describe any methods required to prepare the data for presentation or synthesis, such as handling of missing summary statistics, or data conversions.	117-124
	13c	Describe any methods used to tabulate or visually display results of individual studies and syntheses.	117-124
	13d	Describe any methods used to synthesize results and provide a rationale for the choice(s). If meta-analysis was performed, describe the model(s), method(s) to identify the presence and extent of statistical heterogeneity, and software package(s) used.	117-124
	13e	Describe any methods used to explore possible causes of heterogeneity among study results (e.g. subgroup analysis, meta-regression).	117-124
	13f	Describe any sensitivity analyses conducted to assess robustness of the synthesized results.	124-125
Reporting bias assessment	14	Describe any methods used to assess risk of bias due to missing results in a synthesis (arising from reporting biases).	114-125

Section and Topic	Item #	Checklist item	Location where item is reported
Certainty assessment	15	Describe any methods used to assess certainty (or confidence) in the body of evidence for an outcome.	117-125
**RESULTS**			
Study selection	16a	Describe the results of the search and selection process, from the number of records identified in the search to the number of studies included in the review, ideally using a flow diagram.	126-134
	16b	Cite studies that might appear to meet the inclusion criteria, but which were excluded, and explain why they were excluded.	127-133
Study characteristics	17	Cite each included study and present its characteristics.	Table 1
Risk of bias in studies	18	Present assessments of risk of bias for each included study.	145-186
Results of individual studies	19	For all outcomes, present, for each study: (a) summary statistics for each group (where appropriate) and (b) an effect estimate and its precision (e.g. confidence/credible interval), ideally using structured tables or plots.	Table 2
Results of syntheses	20a	For each synthesis, briefly summarise the characteristics and risk of bias among contributing studies.	Table 2
	20b	Present results of all statistical syntheses conducted. If meta-analysis was done, present for each the summary estimate and its precision (e.g. confidence/credible interval) and measures of statistical heterogeneity. If comparing groups, describe the direction of the effect.	Figure 4
	20c	Present results of all investigations of possible causes of heterogeneity among study results.	Figure 4
	20d	Present results of all sensitivity analyses conducted to assess the robustness of the synthesized results.	Figure 3
Reporting biases	21	Present assessments of risk of bias due to missing results (arising from reporting biases) for each synthesis assessed.	245-250
Certainty of evidence	22	Present assessments of certainty (or confidence) in the body of evidence for each outcome assessed.	245-250
**DISCUSSION**			
Discussion	23a	Provide a general interpretation of the results in the context of other evidence.	245-250
	23b	Discuss any limitations of the evidence included in the review.	254-260
	23c	Discuss any limitations of the review processes used.	321-324
	23d	Discuss implications of the results for practice, policy, and future research.	261-265
**OTHER INFORMATION**			
Registration and protocol	24a	Provide registration information for the review, including register name and registration number, or state that the review was not registered.	70-71
	24b	Indicate where the review protocol can be accessed, or state that a protocol was not prepared.	70-71
	24c	Describe and explain any amendments to information provided at registration or in the protocol.	No
Support	25	Describe sources of financial or non-financial support for the review, and the role of the funders or sponsors in the review.	338-341
Competing interests	26	Declare any competing interests of review authors.	There are no conflicts of interest
Availability of data, code and other materials	27	Report which of the following are publicly available and where they can be found: template data collection forms; data extracted from included studies; data used for all analyses; analytic code; any other materials used in the review.	Data extracted from included studies are publicly available, data used for all analyses; all

Section and Topic	Item #	Checklist item	Location where item is reported
			analytical code in the article

From: Page MJ, McKenzie JE, Bossuyt PM, Boutron I, Hoffmann TC, Mulrow CD, et al. The PRISMA 2020 statement: an updated guideline for reporting systematic reviews. BMJ 2021;372:n71. doi: 10.1136/bmj.n71. This work is licensed under CC BY 4.0. To view a copy of this license, visit https://creativecommons.org/licenses/by/4.0/

Supplementary 2. Peer Review of Electronic Search Strategies

PRESS Guideline — Search Submission & Peer Review Assessment SEARCH

SUBMISSION: THIS SECTION TO BE FILLED IN BY THE SEARCHER

Searcher: Lopes et al.	Email: silvia.lopes.nut@hotmail.com
Date submitted: 24.08.2024	Date requested by: *[Maximum = 5 working days]*

Systematic Review Title:

Nutritional status of iodine and association with iron, selenium and zinc in population studies: a systematic review and meta-analysis

This search strategy is…

	My PRIMARY (core) database strategy — First time submitting a strategy forsearch question and database
X	My PRIMARY (core) strategy — Follow-up review NOT the first time submitting astrategy for search question and database. If this is a response to peer review, itemize the changes made to the review suggestions
	SECONDARY search strategy— First time submitting a strategy for search question and database
	SECONDARY search strategy — NOT the first time submitting a strategy for search question and database. If this is a response to peer review, itemize the changes made to the review suggestions

Database

(i.e., MEDLINE, CINAHL…): *[mandatory]*

MEDLINE

Interface

(i.e., Ovid, EBSCO…): *[mandatory]*

PUBMED

Research Question

(Describe the purpose of the search) *[mandatory]*

Estado nutricional de iodo está diretamente associada ao estado nutricional de ferro, zinco e selênio em estudos populacionais?

PECO Format

(Outline the PICOs for your question — i.e., Patient, Intervention, Comparison, Outcome, and Study Design — as applicable)

**P**	Children, Adolescents, Adults and Elderly (descriptors and terms not used in the search)
**E**	Iodine nutritional status
**C**	No change in iodine nutritional status
**O**	Nutritional status of iron, zinc and selenium

Inclusion Criteria

(List criteria such as age groups, study designs, etc., to be included) *[optional]*

Studies with humans;

Studies that investigated the association between the nutritional status of iodine and the nutritional status of iron, zinc and selenium with the aid of biochemical assessment;

Studies conducted in any date, language or location.

Exclusion Criteria

(List criteria such as study designs, date limits, etc., to be excluded) *[optional]*

- Review studies, letters to the editors, qualitative analyses, case studies or book chapters;

- Work with pregnant women, genetic diseases, such as: Down syndrome and sickle cell anemia; HIV and cancer.

Was a search filter applied?

Yes x No □

If YES, which one(s) (e.g., Cochrane RCT filter, PubMed Clinical Queries filter)? Provide the source if this is a published filter.
[mandatory if YES to previousquestion — textbox]

Não

Other notes or comments you feel would be useful for the peer reviewer? ***[optional]***

Não

Please copy and paste your search strategy here, exactly as run, including the number ofhits per line. ***[mandatory]***

(Add more space, as necessary.)

	**DECS e sinônimos**	**STRATEGY LINES**	**NUMBER OF LOCALIZED STUDIES**
**P**			
	**AND**		
**E**	Iodine Deficiency<br>Iodine Deficiency Disorder<br>Iodine Deficiency, Primary<br>Hypothyroidism, Congenital<br>Cretinism<br>Endemic Cretinism<br>Cretinism, Endemic<br>Fetal Iodine Deficiency Disorder<br>Myxedema, Congenital	((((((((Iodine Deficiency[Title/Abstract]) OR (Iodine Deficiency Disorder[Title/Abstract])) OR (Iodine Deficiency, Primary[Title/Abstract])) OR (Hypothyroidism, Congenital[Title/Abstract])) OR (Cretinism[Title/Abstract])) OR (Endemic Cretinism[Title/Abstract])) OR (Cretinism, Endemic[Title/Abstract])) OR (Fetal Iodine Deficiency Disorder[Title/Abstract])) OR (Myxedema, Congenital[Title/Abstract]))	4862 results
**C**	Não é necessário incluir descritores.		
	**AND**		
**O**	Famine, Occult<br>Iron<br>Iron-56<br>Iron 56<br>Iron Deficiency<br>Anemia, Iron-Deficiency<br>Anemia, Iron Deficiency<br>Iron-Deficiency Anemia<br>Iron Deficiency Anemia<br>Iron-Deficiency Anemias<br>Iron Deficiency Anemias<br>Anemias, Iron-Deficiency<br>Anemias, Iron Deficiency<br>Zinc Deficiency<br>Selenium<br>Selenium-80<br>Selenium 80<br>Growth Disorders<br>Zinc/deficiency<br>Malnutrition<br>Nutritional Deficiency<br>Nutritional Deficiencies<br>Undernutrition<br>Malnourishment	((((((((((((((((((((((((((((((((Famine, Occult[Title/Abstract]) OR (Iron[Title/Abstract])) OR (Iron-56[Title/Abstract])) OR (Iron 56[Title/Abstract])) OR (Iron Deficiency[Title/Abstract])) OR (Anemia, Iron-Deficiency[Title/Abstract])) OR (Anemia, Iron Deficiency[Title/Abstract])) OR (Iron-Deficiency Anemia[Title/Abstract])) OR (Iron Deficiency Anemia[Title/Abstract])) OR (Iron-Deficiency Anemias[Title/Abstract])) OR (Iron Deficiency Anemias[Title/Abstract])) OR (Anemias, Iron-Deficiency[Title/Abstract])) OR (Anemias, Iron Deficiency[Title/Abstract])) OR (Vitamin B 12 Deficiency[Title/Abstract])) OR (Deficiencies, Vitamin B12[Title/Abstract])) OR (Deficiency, Vitamin B 12[Title/Abstract])) OR (Deficiency, Vitamin B12[Title/Abstract])) OR (Vitamin B12 Deficiencies[Title/Abstract])) OR (Vitamin B12 Deficiency[Title/Abstract])) OR (Deficiency Vitamin A[Title/Abstract])) OR (Deficiencies, Vitamin A[Title/Abstract])) OR (Deficiency, Vitamin A[Title/Abstract])) OR (Vitamin A Deficiencies[Title/Abstract])) OR (Zinc Deficiency[Title/Abstract])) OR (Selenium[Title/Abstract])) OR (Selenium-	288591 results

	Malnourishments	80[Title/Abstract])) OR (Selenium 80[Title/Abstract])) OR (Growth Disorders[Title/Abstract])) OR (Zinc/deficiency[Title/Abstract])) OR (Malnutrition[Title/Abstract])) OR (Nutritional Deficiency[Title/Abstract])) OR (Nutritional Deficiencies[Title/Abstract])) OR (Undernutrition[Title/Abstract])) OR (Malnourishment[Title/Abstract])	

*Utilize as adaptações do acrônimo conforme a necessidade.

Default search strategy: Medline database (via Pubmed): (((((((((Iodine Deficiency[Title/Abstract]) OR (Iodine Deficiency Disorder[Title/Abstract])) OR (Iodine Deficiency, Primary[Title/Abstract])) OR (Hypothyroidism, Congenital[Title/Abstract])) OR (Cretinism[Title/Abstract])) OR (Endemic Cretinism[Title/Abstract])) OR (Cretinism, Endemic[Title/Abstract])) OR (Fetal Iodine Deficiency Disorder[Title/Abstract])) OR (Myxedema, Congenital[Title/Abstract]))) AND (((((((((((((((((((((((Famine, Occult[Title/Abstract]) OR (Iron[Title/Abstract])) OR (Iron-56[Title/Abstract])) OR (Iron 56[Title/Abstract])) OR (Iron Deficiency[Title/Abstract])) OR (Anemia, Iron-Deficiency[Title/Abstract])) OR (Anemia, Iron Deficiency[Title/Abstract])) OR (Iron-Deficiency Anemia[Title/Abstract])) OR (Iron Deficiency Anemia[Title/Abstract])) OR (Iron-Deficiency Anemias[Title/Abstract])) OR (Iron Deficiency Anemias[Title/Abstract])) OR (Anemias, Iron-Deficiency[Title/Abstract])) OR (Anemias, Iron Deficiency[Title/Abstract])) OR (Zinc Deficiency[Title/Abstract])) OR (Selenium[Title/Abstract])) OR (Selenium-80[Title/Abstract])) OR (Selenium 80[Title/Abstract])) OR (Growth Disorders[Title/Abstract])) OR (Zinc/deficiency[Title/Abstract])) OR (Malnutrition[Title/Abstract])) OR (Nutritional Deficiency[Title/Abstract])) OR (Nutritional Deficiencies[Title/Abstract])) OR (Undernutrition[Title/Abstract])) OR (Malnourishment[Title/Abstract]))

Supplementary 3. Search strategies in the databases used – 2025-08-02

DATABASE	STRATEGY 1	NUMBER OF STUDIES FOUND
Medline/Pubmed	((((((((((Iodine Deficiency[Title/Abstract]) OR (Iodine Deficiency Disorder[Title/Abstract])) OR (Iodine Deficiency, Primary[Title/Abstract])) OR (Hypothyroidism, Congenital[Title/Abstract])) OR (Cretinism[Title/Abstract])) OR (Endemic Cretinism[Title/Abstract])) OR (Cretinism, Endemic[Title/Abstract])) OR (Fetal Iodine Deficiency Disorder[Title/Abstract])) OR (Myxedema, Congenital[Title/Abstract]))) AND ((((((((((((((((((((((((Famine, Occult[Title/Abstract]) OR (Iron[Title/Abstract])) OR (Iron-56[Title/Abstract])) OR (Iron 56[Title/Abstract])) OR (Iron Deficiency[Title/Abstract])) OR (Anemia, Iron-Deficiency[Title/Abstract])) OR (Anemia, Iron Deficiency[Title/Abstract])) OR (Iron-Deficiency Anemia[Title/Abstract])) OR (Iron Deficiency Anemia[Title/Abstract])) OR (Iron-Deficiency Anemias[Title/Abstract])) OR (Iron Deficiency Anemias[Title/Abstract])) OR (Anemias, Iron-Deficiency[Title/Abstract])) OR (Anemias, Iron Deficiency[Title/Abstract])) OR (Zinc Deficiency[Title/Abstract])) OR (Selenium[Title/Abstract])) OR (Selenium-80[Title/Abstract])) OR (Selenium 80[Title/Abstract])) OR (Growth Disorders[Title/Abstract])) OR (Zinc/deficiency[Title/Abstract])) OR (Malnutrition[Title/Abstract])) OR (Nutritional Deficiency[Title/Abstract])) OR (Nutritional Deficiencies[Title/Abstract])) OR (Undernutrition[Title/Abstract])) OR (Malnourishment[Title/Abstract]))	426 results
Web of science	#1 ((((((((TS=(Iodine Deficiency)) OR TS=(Iodine Deficiency Disorder)) OR TS=(Iodine Deficiency, Primary)) OR TS=(Hypothyroidism, Congenital)) OR TS=(Cretinism)) OR TS=(Endemic Cretinism)) OR TS=(Cretinism, Endemic)) OR TS=(Fetal Iodine Deficiency Disorder)) OR TS=(Myxedema, Congenital)<br><br>#2 (((((((((((((((((((((((TS=(Famine, Occult)) OR TS=(Iron)) OR TS=(Iron-56)) OR TS=(Iron 56)) OR TS=(Iron Deficiency)) OR TS=(Anemia, Iron-Deficiency)) OR TS=(Anemia, Iron Deficiency)) OR TS=(Iron-Deficiency Anemia)) OR TS=(Iron Deficiency Anemia)) OR TS=(Iron-Deficiency Anemias)) OR TS=(Iron Deficiency Anemias)) OR TS=(Anemias, Iron-Deficiency)) OR TS=(Anemias, Iron Deficiency)) OR TS=(Zinc Deficiency)) OR TS=(Selenium)) OR TS=(Selenium-80)) OR TS=(Selenium 80)) OR TS=(Growth Disorders)) OR TS=(Zinc/deficiency)) OR TS=(Malnutrition)) OR TS=(Nutritional Deficiency)) OR TS=(Nutritional Deficiencies)) OR TS=(Undernutrition)) OR TS=(Malnourishment)<br><br>(#1) AND #2	1857 results
CINAHL	S1 - AB Iodine Deficiency OR AB Iodine Deficiency Disorder OR AB Iodine Deficiency, Primary OR AB Hypothyroidism, Congenital OR AB Cretinism OR AB Endemic Cretinism OR AB Fetal Iodine Deficiency Disorder OR AB Myxedema, Congenital<br><br>S2 - AB Famine, Occult OR AB Iron OR AB Iron-56 OR AB Iron 56 OR AB Iron Deficiency OR AB Anemia, Iron-Deficiency OR AB Anemia, Iron	84 results

	Deficiency OR AB Iron-Deficiency Anemia OR AB Iron Deficiency Anemia OR AB Iron-Deficiency Anemias OR AB Iron Deficiency Anemias OR AB Anemias, Iron-Deficiency<br><br>S3- AB Anemias, Iron Deficiency OR AB Zinc Deficiency OR AB selenium OR AB Selenium-80 OR AB Selenium 80 OR AB Growth Disorders OR AB Zinc/deficiency OR AB malnutrition OR AB Nutritional Deficiency OR AB Nutritional Deficiencies OR AB Undernutrition OR AB Malnourishment<br><br>S1 AND (S2 OR S3)	

Supplementary 4. Risk of bias for each individual study assessed by Joanna Briggs Institute critical appraisal checklist for studies

"Checklist for Analytical Cross Sectional Studies", "Checklist for Case Control Studies", "Checklist for Cohort Studies" e "Checklist for Randomized Controlled Trials"

A- Checklist for Analytical Cross Sectional Studies

**Autor/ano**	**Critério**							
	**1**	**2**	**3**	**4**	**5**	**6**	**7**	**8**
Wolde-Gebriel et al., 1993	N	Y	Y	Y	Y	N	N	Y
Kvíčala et al., 1995	Y	Y	Y	Y	Y	Y	Y	Y
Hampel et al., 1997	Y	Y	Y	Y	N	N	Y	Y
Ozata et al., 1999	Y	Y	Y	Y	Y	N	N	Y
Zagrodzki et al., 2000	Y	Y	Y	Y	Y	Y	Y	Y
Erdoğan et al., 2001	Y	Y	Y	Y	Y	Y	Y	Y
Aydin et al., 2002	Y	Y	Y	Y	Y	Y	Y	Y
Azizi et al., 2002	Y	Y	Y	Y	Y	N	N	U
Eftekhari et al., 2006	Y	Y	Y	Y	Y	Y	Y	Y
Thurlow et al., 2006	Y	Y	Y	Y	Y	Y	Y	Y
Dabbaghmanesh et al., 2008	Y	Y	Y	Y	N	N	Y	Y
Zagrodzki; Ratajczak, 2008	U	Y	Y	U	Y	N	N	Y
Doupis et al., 2009	Y	Y	Y	Y	Y	Y	Y	Y
Keshteli et al., 2009	Y	Y	Y	Y	Y	N	N	U
Moaddab et al., 2009	N	Y	Y	Y	Y	Y	Y	Y
Hashemipour et al., 2010	Y	Y	Y	Y	Y	Y	Y	Y
Keshteli et al., 2010	Y	Y	Y	Y	Y	Y	Y	Y
Henjum et al., 2012	Y	Y	Y	Y	Y	Y	Y	Y
Sanjari; Gholamhoseiniana; Nakhaee, 2012	Y	Y	Y	Y	Y	Y	Y	Y
Liu et al., 2013	Y	Y	Y	Y	Y	Y	Y	Y
Yavuz et al., 2014	Y	Y	Y	Y	Y	Y	Y	Y
Khatiwada et al., 2016	Y	Y	Y	Y	Y	Y	Y	Y
Luo et al., 2017	Y	Y	Y	Y	Y	Y	Y	Y
Suhail; Alsel; Batool, 2020	Y	Y	Y	Y	Y	Y	Y	Y
Campos et al., 2021	Y	Y	Y	Y	Y	Y	Y	Y
Islam et al., 2021	Y	Y	Y	Y	Y	Y	Y	Y
Turan; Turksoy, 2021	Y	Y	Y	Y	N	N	Y	Y
Berger et al., 2025	Y	Y	Y	Y	N	Y	Y	Y
TOTAL	25	28	28	27	24	20	23	26

Y = Yes, N = No, U = Unclear, NA = Not applicable; 1= Criteria for inclusion in the sample clearly defined; 2= Study subjects and the setting described in detail; 3= Exposure measured in a valid and reliable way; 4= Objective and standard criteria for measurement; 5= Confounding factors identified; 6= Strategies to deal with confounding factors; 7= Outcomes measured in a valid and reliable way e 8= Appropriate statistical analysis.

B- Checklist for Case Control Studies

**Autor/ano**	**Critério**									
	**1**	**2**	**3**	**4**	**5**	**6**	**7**	**8**	**9**	**10**
Ravaglia *et al.*, 2000	N	Y	Y	Y	Y	N	N	Y	Y	Y
Cinaz *et al.*, 2004	N	N	Y	Y	Y	N	N	Y	Y	Y
Hekimsoy *et al.*, 2004	Y	Y	Y	Y	Y	Y	Y	Y	Y	Y
Kandhro *et al.,* 2008	Y	Y	Y	Y	Y	Y	Y	Y	Y	Y
Kishosha; Galukand; Gakwaya 2011	Y	Y	Y	Y	Y	Y	Y	Y	Y	Y

Çelik *et al.*, 2014	N	Y	Y	Y	Y	N	N	Y	Y	Y
TOTAL	3	5	6	6	6	3	3	6	6	6

Y = Yes, N = No, U = Unclear, NA = Not applicable; 1=Were the groups comparable other than the presence of disease in cases or the absence of disease in controls?; 2=Were cases and controls matched appropriately?; 3 =Were the same criteria used for identification of cases and controls?; 4 =Was exposure measured in a standard, valid and reliable way?; 5 =Was exposure measured in the same way for cases and controls?; 6 = Were confounding factors identified?; 7= Were strategies to deal with confounding factors stated?; 8= Were outcomes assessed in a standard, valid and reliable way for cases and controls?; 9= Was the exposure period of interest long enough to be meaningful?; 10=Was appropriate statistical analysis used?

C- Checklist for Randomized Controlled Trials

**Autor/ano**	**Critério**												
	**1**	**2**	**3**	**4**	**5**	**6**	**7**	**8**	**9**	**10**	**11**	**12**	**13**
Olivieri *et al.*, 1995	Y	Y	Y	Y	Y	U	Y	Y	Y	Y	Y	Y	Y
Olivieri *et al.*, 1996	Y	Y	Y	Y	Y	Y	Y	Y	Y	Y	Y	Y	Y
Gashu *et al.*, 2009	Y	U	Y	Y	Y	Y	Y	Y	Y	Y	Y	Y	Y
TOTAL	3	2	3	3	3	2	3	3	3	3	3	3	3

Y = Yes, N = No, U = Unclear, NA = Not applicable; 1 =Was true randomization used for assignment of participants to treatment groups?; 2=Was allocation to treatment groups concealed?; 3=Were treatment groups similar at the baseline?; 4= Were participants blind to treatment assignment?; 5= Were those delivering treatment blind to treatment assignment?; 6= Were outcomes assessors blind to treatment assignment?; 7=Were treatment groups treated identically other than the intervention of interest?; 8=Was follow up complete and if not, were differences between groups in terms of their follow up adequately described and analyzed?; 9= Were participants analyzed in the groups to which they were randomized?; 10 =Were outcomes measured in the same way for treatment groups?; 11=Were outcomes measured in a reliable way?; 12= Was appropriate statistical analysis used?; 13= Was the trial design appropriate, and any deviations from the standard RCT design (individual randomization, parallel groups) accounted for in the conduct and analysis of the trial?

D- Checklist for Cohort Studies

**Autor/ano**	**Critério**										
	**1**	**2**	**3**	**4**	**5**	**6**	**7**	**8**	**9**	**10**	**11**
Rasmussen *et al.*, 2011	Y	Y	Y	Y	Y	Y	Y	Y	Y	Y	Y
El-masry *et al.,* 2018	Y	Y	Y	Y	Y	Y	Y	Y	Y	Y	Y
Gu *et al.*, 2019	Y	Y	Y	Y	Y	Y	Y	Y	Y	Y	Y
TOTAL	3	3	3	3	3	3	3	3	3	3	3

Y = Yes, N = No, U = Unclear, NA = Not applicable; 1= Were the two groups similar and recruited from the same population?; 2= Were the exposures measured similarly to assign people to both exposed and unexposed groups?; 3=Was the exposure measured in a valid and reliable way?; 4=Were confounding factors identified?; 5=Were strategies to deal with confounding factors stated?; 6= Were the groups/participants free of the outcome at the start of the study (or at the moment of exposure)?; 7=Were the outcomes measured in a valid and reliable way?; 8=Was the follow up time reported and sufficient to be long enough for outcomes to occur?; 9=Was follow up complete, and if not, were the reasons to loss to follow up described and explored?; 10=Were strategies to address incomplete follow up utilized?; 11=Was appropriate statistical analysis used?

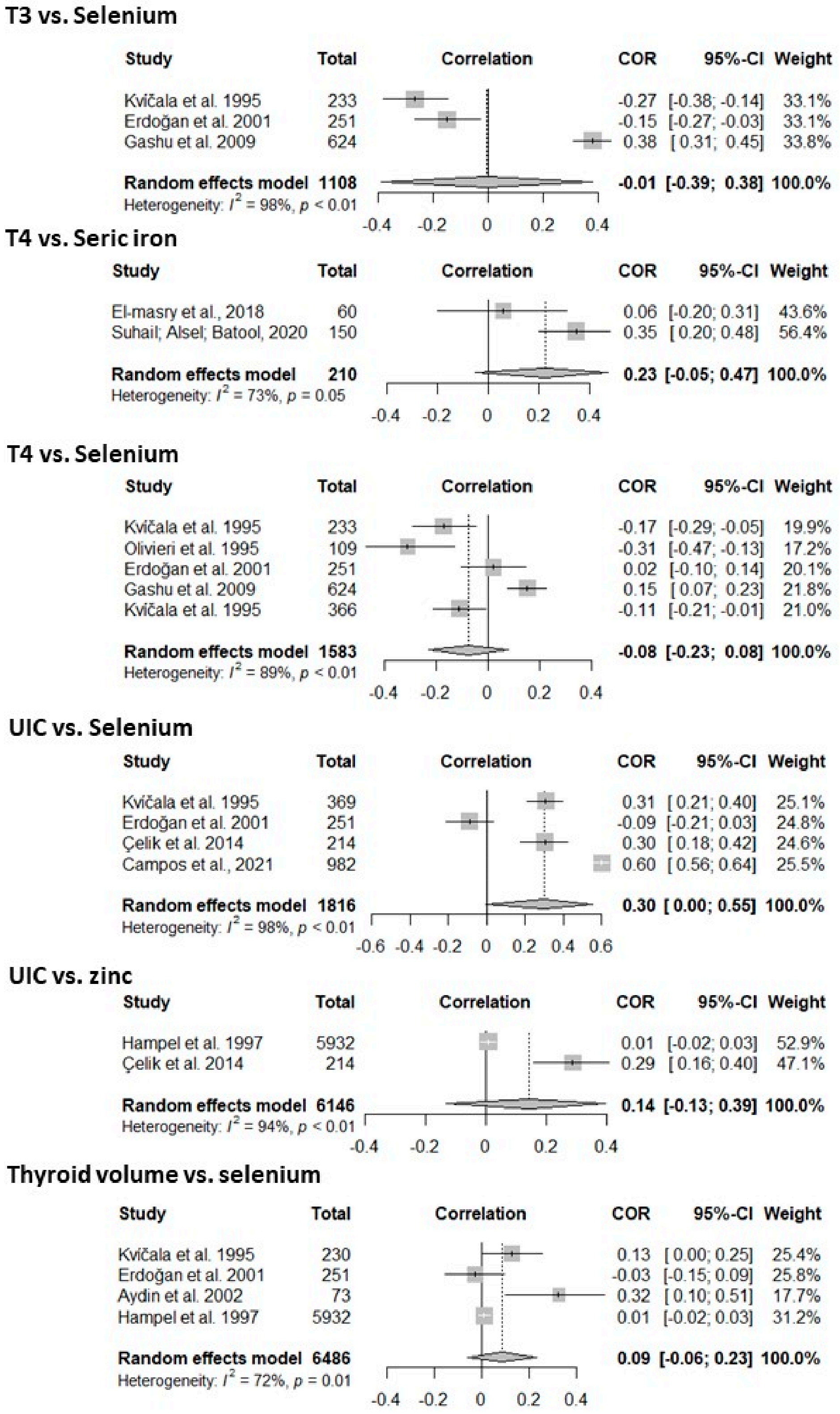

Supplementary figure 1. Meta-analysis of the correlation coefficients between the nutritional status of iodine (T3, T4, TSH, and UIC) and that of iron (serum iron, ferritin and hemoglobin), selenium and zinc.

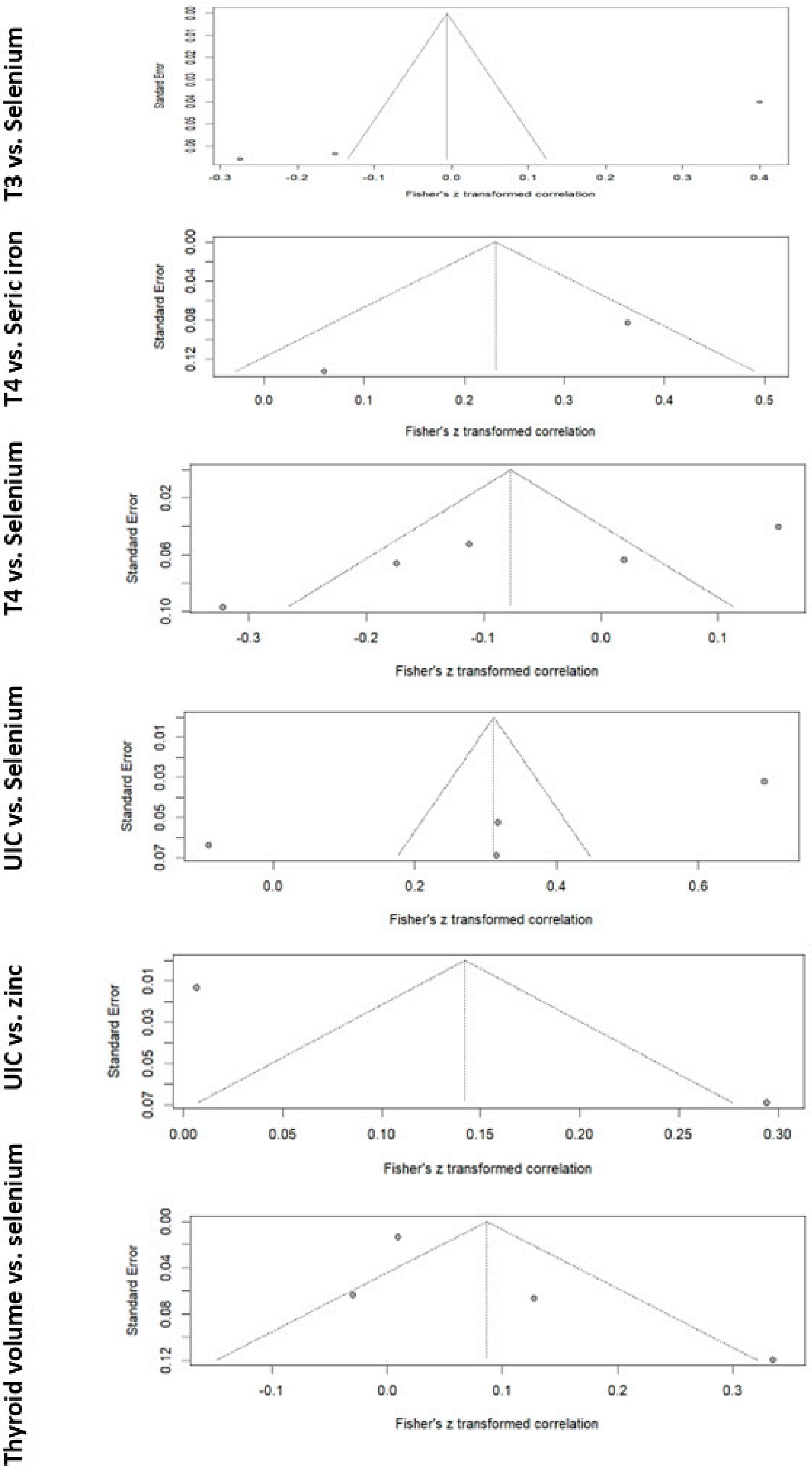
T3 vs. Selenium
T4 vs. Seric iron
T4 vs. Selenium
UIC vs. Selenium
UIC vs. zinc
Thyroid volume vs. selenium
Standard Error
Fisher's z transformed correlation

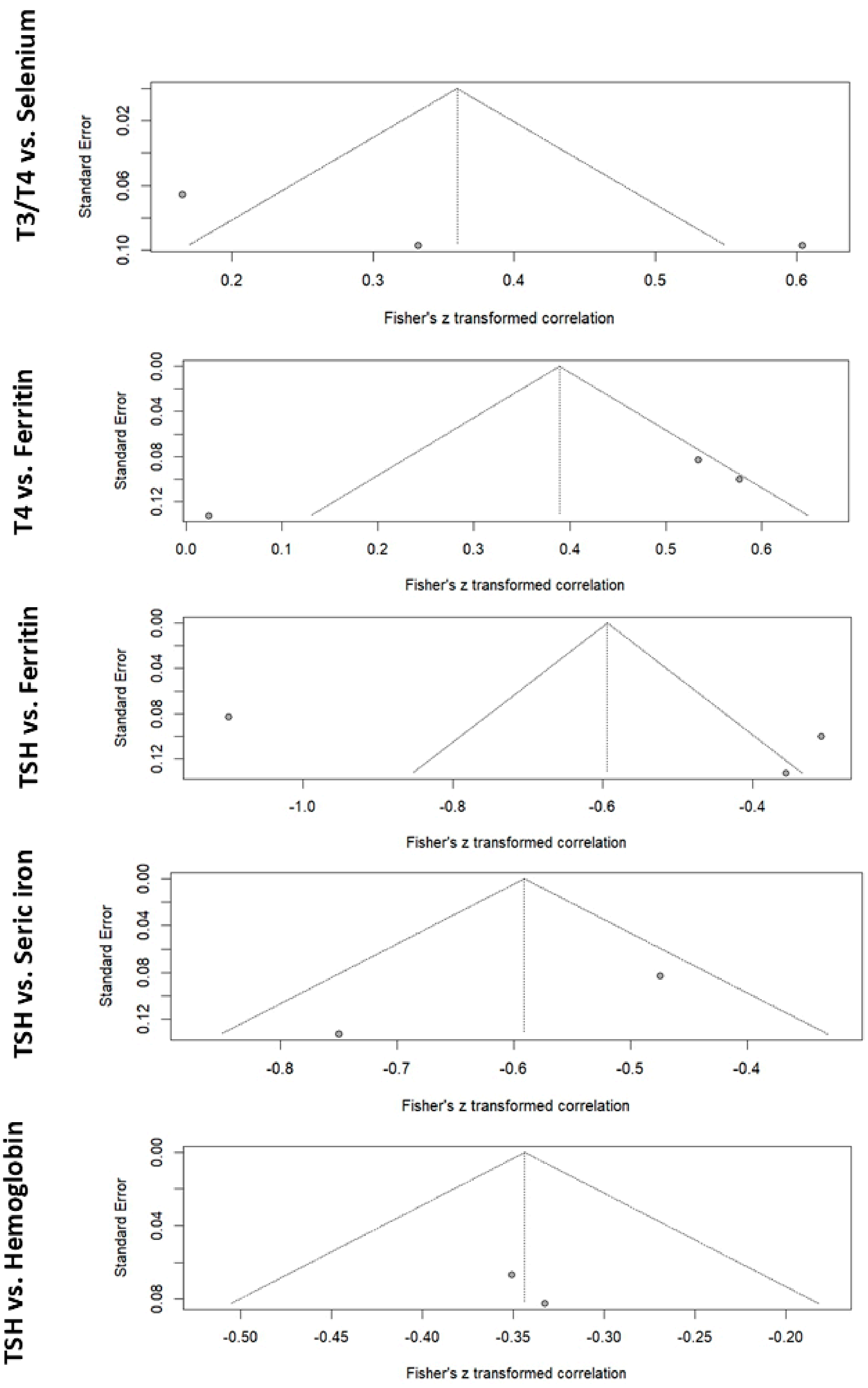

Supplementary figure 2. Funnel plot of meta-analysis of the correlation coefficients between the nutritional status of iodine (T3, T4, TSH, and UIC) and that of iron (serum iron, ferritin and hemoglobin), selenium and zinc.

T3/T4 vs. Selenium

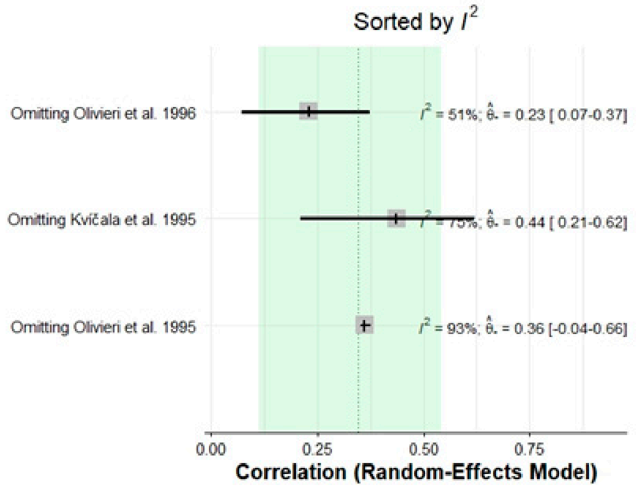

T4 vs. Ferritin

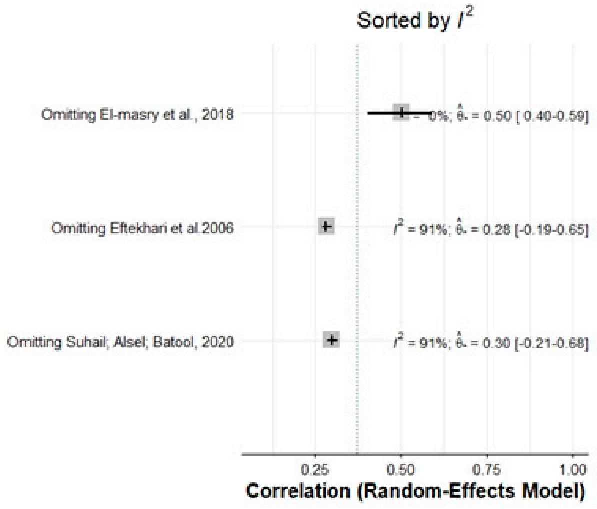

TSH vs. Ferritin

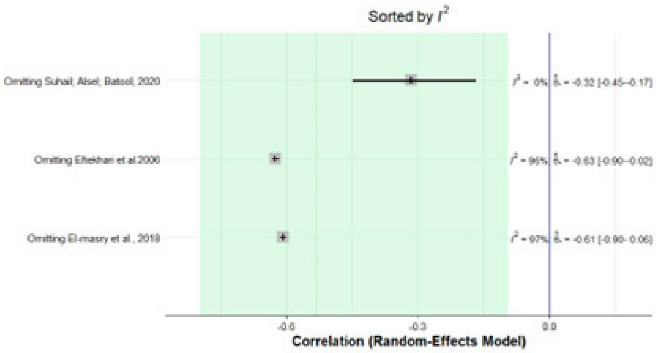

TSH vs. Seric iron

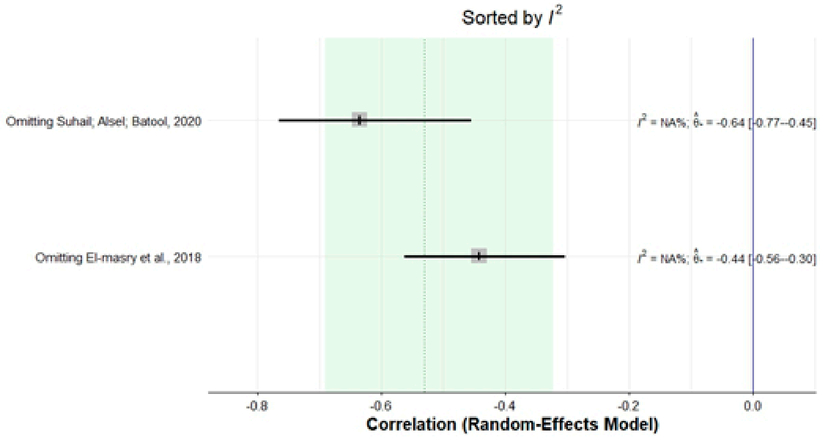

TSH vs. Hemoglobin

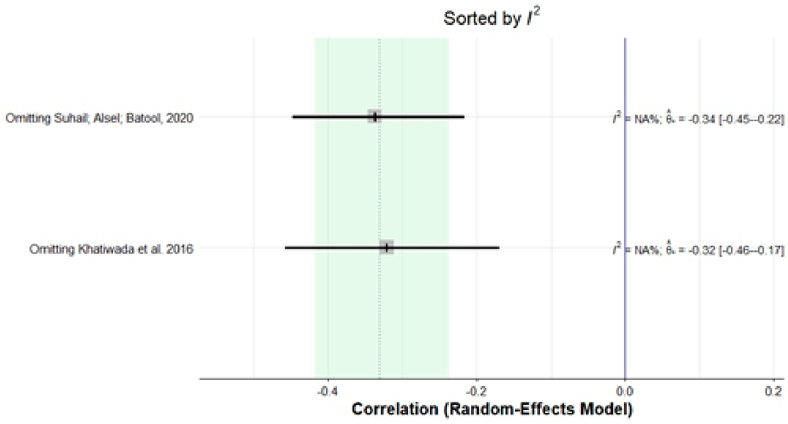

T3 vs. Selenium

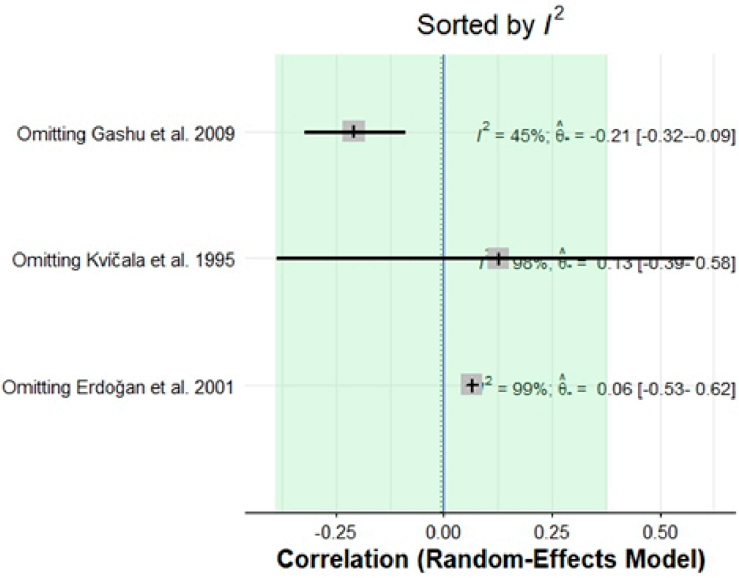

UIC vs. zinc

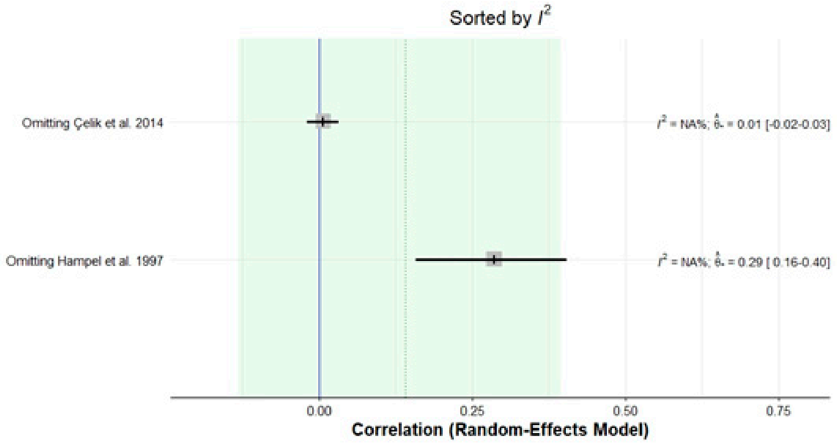

Thyroid volume vs. selenium

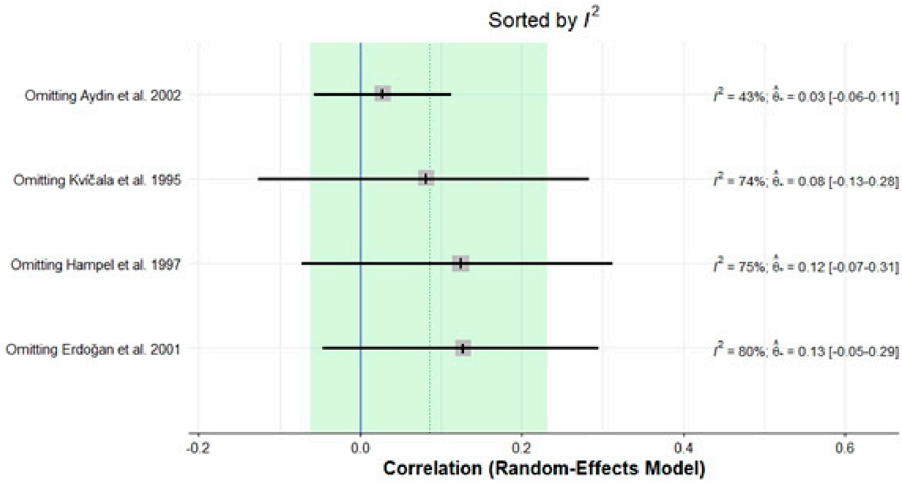

T4 vs. Seric iron

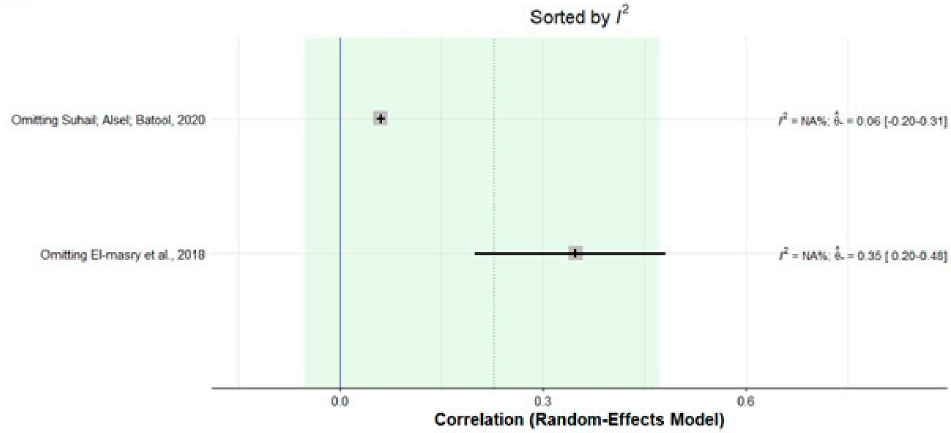

T4 vs. Selenium

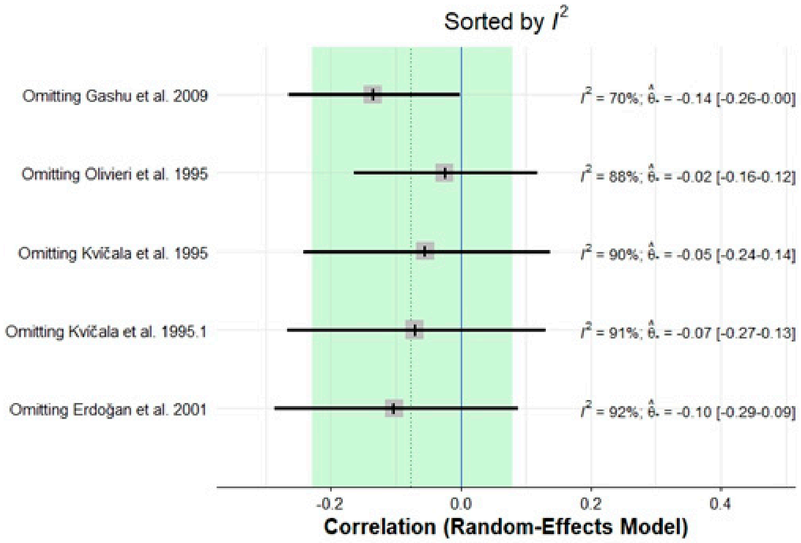

UIC vs. Selenium

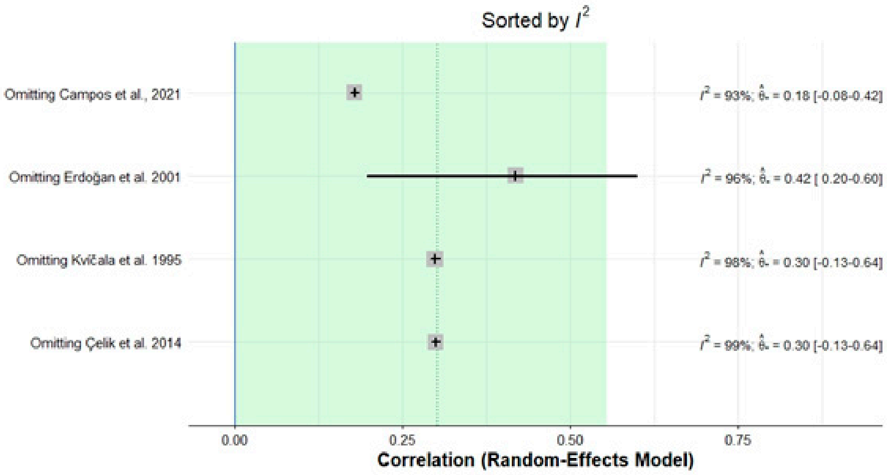

Supplementary figure 3. Influence analysis was applied
